# Low-data interpretable deep learning prediction of antibody viscosity using a biophysically meaningful representation

**DOI:** 10.1038/s41598-023-28841-4

**Published:** 2023-02-20

**Authors:** Brajesh K. Rai, James R. Apgar, Eric M. Bennett

**Affiliations:** 1 Pfizer Worldwide Research Development and Medical, Machine Learning and Computational Sciences, 610 Main Street, Cambridge, MA 02139 USA; 2 Pfizer Worldwide Research Development and Medical, Biomedicine Design, 610 Main Street, Cambridge, MA 02139 USA

**Keywords:** Machine learning, Computational models

## Abstract

Deep learning, aided by the availability of big data sets, has led to substantial advances across many disciplines. However, many scientific problems of practical interest lack sufficiently large datasets amenable to deep learning. Prediction of antibody viscosity is one such problem where deep learning methods have not yet been explored due to the relative scarcity of relevant training data. In this work, we overcome this limitation using a biophysically meaningful representation that enables us to develop generalizable models even under limited training data. We present, PfAbNet-viscosity, a 3D convolutional neural network architecture, to predict high-concentration viscosity of therapeutic antibodies. We show that with the electrostatic potential surface of the antibody variable region as the only input to the network, the models trained on as few as couple dozen datapoints can generalize with high accuracy. Our feature attribution analysis shows that PfAbNet-viscosity has learned key biophysical drivers of viscosity. The applicability of our approach to other biological systems is discussed.

## Introduction

Despite substantial advances across many disciplines, application of deep learning to many real-world scientific problems has been hampered due to insufficient training data and the difficulty in acquiring such data in a timely fashion. The discovery and development of monoclonal antibodies (mAbs)^1,2^, a therapeutic modality for a wide range of diseases and indications, is one such area where deep learning has so far been applied relatively infrequently. Due to the time, material, and other resource constraints associated with experimental measurements, characterization of various mAb developability properties^3^ such as chemical stability^4,5^, clinical immunogenicity, and viscosity has been limited to a small number of candidate molecules.

In this work, we address the constraint of small data in developing predictive models for antibody viscosity, a key developability attribute for mAb-based therapeutics. Viscosity is an important consideration in the development of mAbs because to maintain desired efficacy and avoid the need for frequent dosing, therapeutic antibodies are formulated at high concentrations, aiming to deliver subcutaneously > 100 mg of active ingredients within a small volume (≤ 1 mL)^6^. At such high concentration, antibodies are prone to exhibit high viscosity and can present significant formulation, manufacturing, and administration challenges. Although higher-throughput data collection alternatives such as DLS are available, rheometric measurement of viscosity is a preferred experimental technique. However, rheometric viscosity measurement requires large amounts (> 100 mg) of purified proteins, which are generally not available in the early candidate selection stages, and, therefore, can be carried out only for the most promising molecules in the later stages of the discovery and development pipeline. Therefore, only a limited number of public or proprietary molecules have been experimentally characterized for this important therapeutic property. Consequently, previous publications on this topic have primarily focused on identifying meaningful physicochemical correlates^7–13^ of viscosity, with a few others describing biophysical^14^ or data-driven^9,12,13,15^ models.

Here, we present PfAbNet-viscosity (Pfizer Antibody Network for viscosity; henceforth referred to as PfAbNet in short), a deep learning architecture, which we originally developed to screen mAb candidates for potential viscosity liabilities in the early stages of our antibody therapeutic discovery programs. Using the electrostatic potential (ESP) surface of the antibody variable region (Fv) as the only input, PfAbNet predicts the viscosity of a test antibody in high-concentration solution. We describe the underlying 3D convolutional neural network (3D-CNN), a deep learning technique that has been applied to a wide range of computational chemistry^16–19^ and structural biology^20–22^ problems by leveraging large structural datasets. We present the model training procedure, assess the generalization accuracy of the models, and discuss the insights generated from our feature attribution analysis.

## Results

### PfAbNet: a 3D-convolutional neural network to predict antibody viscosity

The PfAbNet architecture and input representation scheme were developed and refined using our in-house antibodies and viscosity data, all measured under standardized conditions, as described previously^23^. While a subset of these antibodies came from our various mAb therapeutic discovery programs, others were specifically designed as part of an internal effort to generate a robust and chemically diverse dataset to enable development of generalizable predictive models of viscosity.

**Figure 1:**
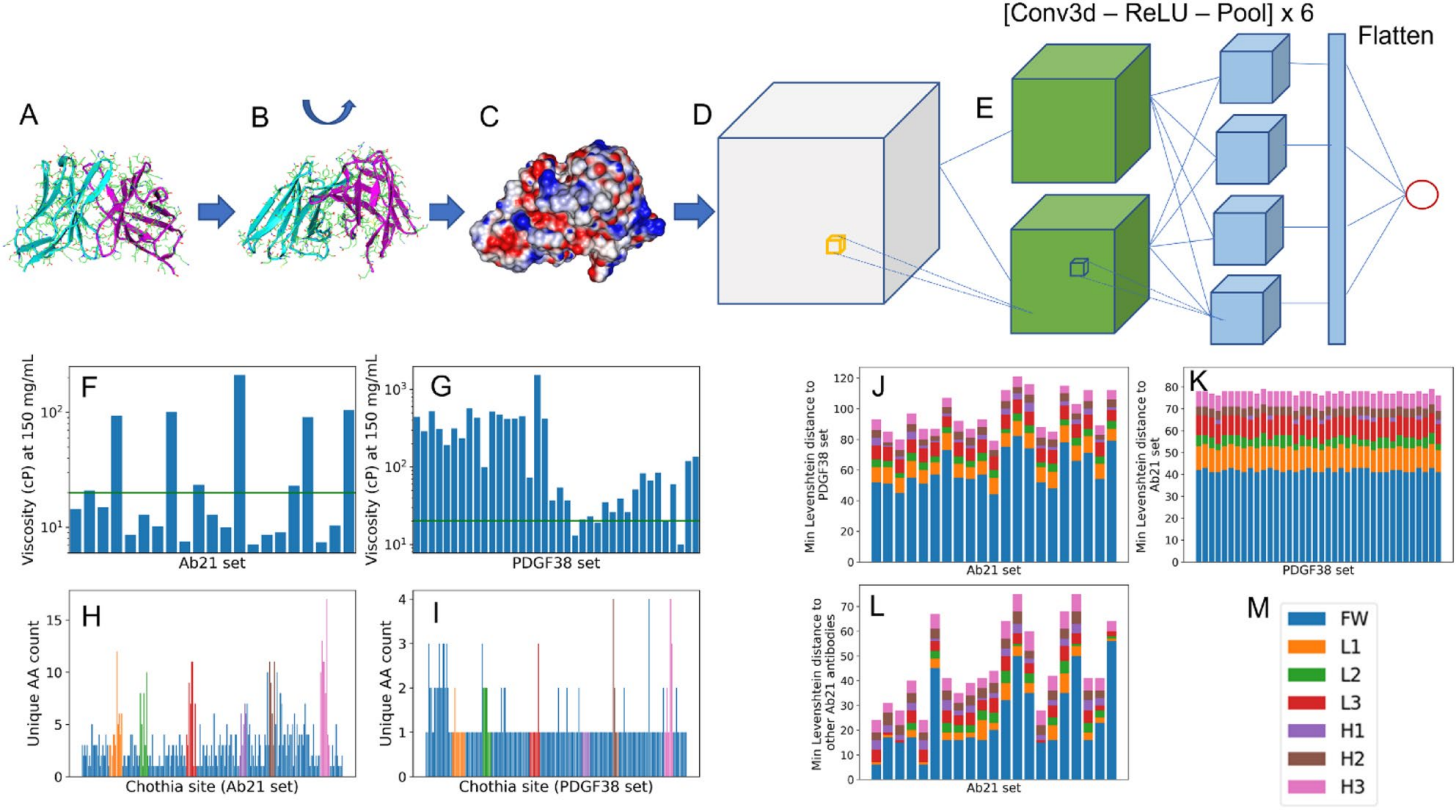
The PfAbNet pipeline and the datasets. (**A**) The starting Fv domain structure or homology model. (**B**) Training data augmentation and inference ensemble generation through random rotation of the starting Fv structure. (**C**) Generation of molecular surface and ESP. (**D**) Cubic grid with ESP surface shell. (**E**) Illustration of the 3D-CNN architecture. (**F**,**G**) Experimental viscosity of the Ab21 (**F**) and PDGF38 (**G**) antibodies at 150 mg/mL concentration. The horizontal line in these panels represent the 20 cP threshold that defines the low- and high-viscosity classes. (**H**,**I**) Amino acid variability in the Ab21 (**H**) and PDGF38 (**I**) datasets at different Chothia positions across the variable region sequence. (**J**–**L**) Minimum Levenshtein distance between the variable region sequence of the Ab21 antibodies with respect to the PDGF38 set (**J**), PDGF38 antibodies with respect to the Ab21 set (**K**), and Ab21 antibodies with respect to the other antibodies in the same set (**L**). (**M**) The coloring scheme used in depicting the contribution from the framework and CDR loop regions to the distributions in (**H**–**L**).

Recognizing the importance of surface charge patches on antibody viscosity from previous studies^10,14,24,25^, we designed our neural network architecture to operate on the ESP surface. We reasoned that a network trained on molecular surface will generalize better compared to a similar network trained on the entire 3D structure input, since a surface representation prevents the model from memorizing less relevant structural details that may not be particularly important for viscosity but could lead to overfitting. With the surface ESP of the given Fv structure as the only input, PfAbNet applies a series of 3D convolution, activation, and pooling operations, transforming the input 3D grid to a numerical value that represents the viscosity (η) of that antibody at 150 mg/mL concentration under previously reported experimental conditions^12,23^ (Fig. 1).

### PfAbNet models show high generalization accuracy in low-data regime

We demonstrate PfAbNet generalization performance by training and evaluating the models using a dataset containing experimental viscosity of 59 IgG1 subtype antibodies. The antibodies in this dataset have been developed against a variety of antigens and comprise 21 FDA-approved drugs^12^ and 38 publicly available antibody variants that were originally developed in a Pfizer-internal program^23^. We trained and evaluated PfAbNet by splitting this dataset into two groups based on the source of these antibodies, generating (1) a small dataset comprising 38 anti-PDGF antibody variants (the PDGF38 set)^23^ and (2) another small but highly diverse dataset comprising 21 FDA-approved antibody therapeutics (the Ab21 set)^12^. As shown in Fig. 1F,G, the antibodies in these two test sets, though each strongly biased towards the opposite ends of the viscosity distribution, span a wide range of viscosity at 150 mg/mL concentration. The Fv sequences in the two test sets show variability at a large number of Chothia^26^ sites across the framework and CDR regions (Fig. 1H,I). We also note significant variability, both with in Ab21 as well as across the two sets (Fig. 1J–L), with very high minimum Levenshtein distances (20–70 with in the Ab21 set and 80–120 between Ab21 and PDGF38). Given such large diversity of our datasets, we believe that our data splitting approach will provide an accurate assessment of PfAbNet generalization performance.

A different group of 8 IgG1 antibodies from a previous publication^27^ was included as an additional test set (6 of the 14 antibodies in this study are already included in Ab21; we refer to the remaining unique 8 antibodies as Ab8 set). While Ab8 sequences are highly diverse and show variability across a large number of Chothia sites, due to the small size and a narrow viscosity range (Fig. S1), this dataset alone is not suitable for either training or evaluation. However, together with the PDGF38 antibodies, the Ab8 set can be used to further validate PfAbNet, as we show later.

The network was trained from scratch, generating two separate PfAbNet models that we refer to as: (1) PfAbNet-PDGF38 (trained on the PDGF38 set) and (2) PfAbNet-Ab21 (trained on the Ab21 set). We trained additional models, referred to as PfAbNet-LOOCV, to test leave-one-out cross-validation performance, where each Ab21 antibody is left-out once as the test set while the model is trained on the remaining 58 antibodies (38 from the PDGF38 and 20 from the Ab21 set; see Methods).

**Figure 2:**
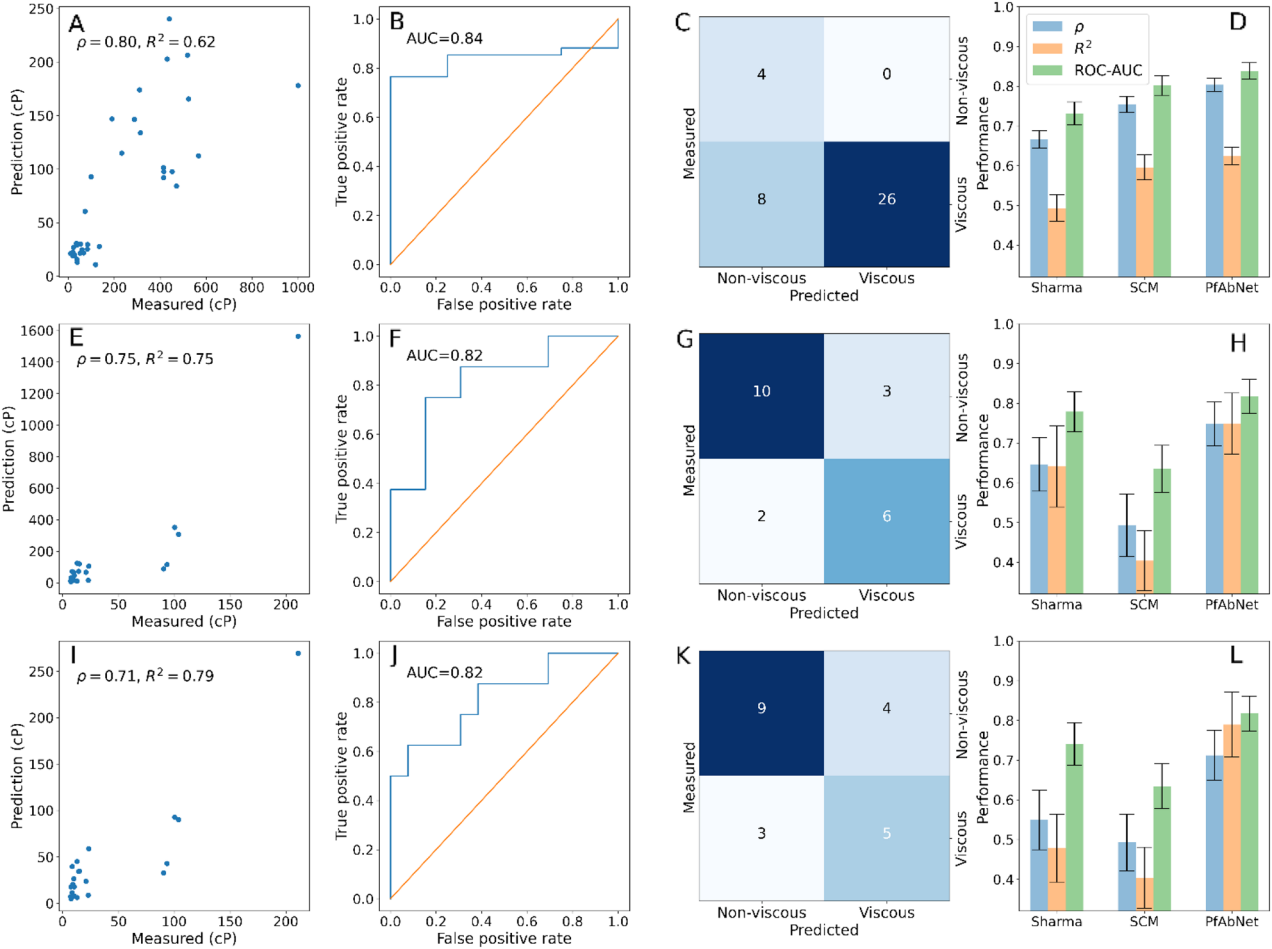
Performance of PfAbNet and previous sequence- and structure-based methods. All predictions and experimental values correspond to viscosity at 150 mg/mL concentration. (**A**) PfAbNet-Ab21 predictions for the PDGF38 antibodies. (**B**,**C**) Classification performance of PfAbNet-Ab21 on the PDGF38 test set: ROC curve (**B**) and confusion matrix (**C**). (**D**) Performance of PfAbNet-Ab21 and previous methods (re-trained Sharma model and SCM) on the PDGF38 test set based on Spearman rank-order correlation, R^2^, and ROC-AUC metrics. (Middle row) The performance of PfAbNet-PDGF and previous methods on the Ab21 test set: (**E)** PfAbNet-PDGF prediction vs experimental viscosity, (**F**) classification performance using ROC curve, (**G**) confusion matrix, and (**H**) Spearman rank-order correlations, R^2^, and ROC-AUC. (Bottom row) The leave-one-out performance of the PfAbNet, SCM, and re-trained Sharma models on the Ab21 test set: (**I)** PfAbNet-LOOCV prediction vs experimental viscosity, (**J**,**K**) classification performance shown using ROC curve (**J**) and confusion matrix (**K**), (**L**) Spearman rank-order correlation, R^2^, and ROC-AUC. The error bars represent the 95% confidence interval estimated with 500 bootstrap samples. Each confusion matrix was calculated using the optimal operating point, derived from the corresponding ROC curve, as the cutoff for viscous vs. non-viscous class.

The performance of PfAbNet-Ab21 models were evaluated on the corresponding held-out test set, PDGF38 and a combined test set comprising PDGF38 and Ab8 antibodies. Whereas the performance of PfAbNet-PDGF38 and PfAbNet-LOOCV models were evaluated on the same set of Ab21 antibodies (Fig. 2, see “Methods”). Remarkably, despite the small amount of training data and low sequence similarity between the training and the test sets, these models produced high Spearman rank-order correlation and R^2^ between the predicted and experimental viscosity (Fig. 2A,E,I, Fig. S2A). PfAbNet predictions for the test set antibodies are provided in Tables S1, S2, and S3.

We further tested the ability of PfAbNet models to discriminate between high and low viscosity antibodies. The classification performance was assessed using Receiver Operating Characteristic (ROC) curve and the associated Area Under the Curve (AUC). We used 20 centipoise (cP) as the threshold to define two classes: viscous (> = 20 cP) and non-viscous (< 20 cP). The 20 cP cutoff was chosen because high concentration solutions of mAbs with viscosity near this threshold are known to present formulation, manufacturing, and administration challenges^2,6^. As illustrated by the ROC plots in Fig. 2B,F,J, and Fig. S2B, all three models can reliably distinguish between viscous and non-viscous antibodies in the corresponding left-out test sets. While the classification performance of LOOCV and PfAbNet-PDGF models, as measured by ROC-AUC, are identical (AUC = 0.82), the LOOCV models, which were trained by incorporating fewer than two dozen additional data points, can recover true positives more efficiently compared to the PfAbNet-PDGF model. For example, the LOOCV models were able to retrieve over 50% true positives (Fig. 2J) compared to < 40% (Fig. 2F) that could be retrieved by the PfAbNet-PDGF model before these models made any false positive prediction.

Despite the strong correlation shown in Fig. 2, the PfAbNet models either systematically underestimate (PfAbNet-Ab21) or overestimate (PfAbNet-PDGF) the experimental values, primarily due the skewed distribution of the measured viscosity in each training set. Therefore, the optimal operating point (OOP) on the ROC curve, which defines the most appropriate cutoff to discriminate between the two viscosity classes, vary significantly between different PfAbNet models (22 cP for PfAbNet-Ab21, 72 cP for PfAbNet-PDGF, and 33 cP for PfAbNet-LOOCV). Nonetheless, the confusion matrix generated using the OOP-based cutoff for each PfAbNet model correctly identifies the majority of the low and high viscosity antibodies in the corresponding test set (Fig. 2C,G,K).

We further compared the classification accuracy of PfAbNet-Ab21 and PfAbNet-PDGF models against a null model that assigns all test set antibodies to a single viscosity class (either low- or high-viscosity), corresponding to the majority class represented in that set. As shown in Fig. S3, PfAbNet-PDGF outperforms the null model (classification accuracy, 76% ± 4% vs. 62% ± 5%,), even though the Ab21 test set is slightly skewed towards the low-viscosity class (13 out of 21). Since the PDGF38 test set exhibits an even greater imbalance (34 of the 38 antibodies are from the high-viscosity class) and thus highly favorable to the null model used here, PfAbNet-Ab21 underperforms the null model (classification accuracy, 82% ± 2% vs 89% ± 2%). On a combined test set, comprising PDGF38 and Ab8 antibodies, PfAbNet-Ab21 marginally outperforms the null model (classification accuracy 80% ± 2% vs 78% ± 2%), since incorporating Ab8 antibodies reduces the test set imbalance.

PfAbNet-Ab21 classification accuracy relative to the null model improves systematically as test sets get more balanced in their composition of low- and high-viscosity antibodies. This is illustrated in Fig. S3 (bars D and E) using two groups of test sets, each generated with different levels of down sampling of the high-viscosity class in the combined PDGF38 and Ab8 set (see Supplementary Note 1). These additional evaluations show that the gap between the classification accuracy of PfAbNet-Ab21 and null model increases as the test sets become more balanced (82% ± 3% vs 67% ± 3% on a test set with 2:1 ratio of high- to low-viscosity antibodies and 80% ± 4% vs 50% ± 5% on a test set comprising equal number of high- and low-viscosity antibodies).

### Comparison with previous methods

We next compared PfAbNet with two previously reported methods: Sharma model^15^ and Surface Charge Model (SCM)^14^. These methods were chosen because they differ significantly in their choice of features (sequence- vs structure-based) and modeling (data driven vs biophysical) approach. While the Sharma model was derived from a linear regression over three sequence-derived features (Fv net charge, VL-VH charge asymmetry, and hydrophobicity), SCM is a structure-based, non-parametric biophysical model that quantifies negative charge distribution over Fv surfaces to predict antibody viscosity at 150 mg/mL concentration.

The predictions based on the Sharma model with default parameters, as reported in the original publication, were significantly off from the actual measurements and showed negative correlation with experimental data from both the PDGF38 and Ab21 test sets. This was not unexpected, since the original parameters were derived by fitting to measured viscosity at 180 mg/mL concentration from a set of 14 therapeutic antibodies that likely covered a very different sequence space than the antibodies studied in this work. Therefore, we re-trained the Sharma model separately using the PDGF38, Ab21, and LOOCV training set following the same dataset split procedure we used to train and evaluate the PfAbNet models (PfAbNet-PDGF38, PfAbNet-Ab21, and PfAbNet-LOOCV). Each resulting model was then used to predict viscosity of corresponding left-out test set antibodies. Since SCM does not have any adjustable parameters, it was not necessary to re-train this model for each specific dataset. We used the previously reported SCM predictions for the PDGF38^23^ and Ab21^12^ antibodies for the model comparisons presented here.

As shown in Fig. 2, Table 1, and Table S4, PfAbNet achieves significantly better performance compared to the two baseline methods on each test set, both in the regression and the classification settings.

**Table 1:** Performance of PfAbNet and the baseline models.

Training set (N)	Test set (N)	Spearman rank-order correlation^^^^					ROC-AUC^^^^				
		Sharma^^^	SCM	PfAbNet	p-value		Sharma^^^	SCM	PfAbNet	p-value	
					PfAbNet vs. Sharma	PfAbNet vs. SCM				PfAbNet vs. Sharma	PfAbNet vs. SCM
Ab21 (21)	PDGF38 (38)	0.67 (0.02)	0.75 (0.02)	0.80 (0.02)	7e−174	1e−37	0.73 (0.03)	0.80 (0.03)	0.84 (0.02)	2e−84	5e−18
PDGF38 (38)	Ab21 (21)	0.65 (0.07)	0.49 (0.07)	0.75 (0.06)	2e−27	6e−118	0.78 (0.05)	0.63 (0.06)	0.82 (0.04)	1e−6	8e−96
PDGF38 + Ab21 (LOOCV) (58)	Ab21 (21)	0.55 (0.07)	0.49 (0.08)	0.71 (0.06)	4e−54	5e−72	0.74 (0.06)	0.63 (0.06)	0.82 (0.04)	1e−25	2e−81

Regression performance based on R^2^ are shown in Table S4.

^^^Performance on each test set was evaluated using parameters that were derived by fitting to the corresponding training set data.

^^^^values in parentheses represent the 95% confidence interval based on bootstrap standard error.

In our internal validations using a larger set of antibodies that were separated into training and test sets based on therapeutic programs (and as a consequence grouped by sequence families), PfAbNet showed similar ability to rank-order and distinguish between low- and high-viscosity antibodies (data not shown). The PfAbNet performance presented here is particularly remarkable since the training and test set antibodies share little sequence similarity and were developed against very different antigen targets (Fig. 1J–L). Thus, these results suggest that PfAbNet can be an effective tool to screen and select mAb candidates with desirable viscosity characteristics.

### PfAbNet interpretability assessment using surface feature attribution

We analyzed the trained networks to understand the patterns our models have learnt. We used Integrated Gradients^28^, a deep learning model interpretability technique. Given a trained PfAbNet model and an ESP grid, this method was used to assign an attribution score for each input grid point that quantifies how important that grid point is to the predicted viscosity. A grid point with a positive attribution score indicates that the underlying structural feature contributes, according to the model, to an increase in viscosity. Conversely, a motif that overlaps with grid points with negative attribution score reduces viscosity. To simplify the analysis, we focus on a subset of “significant attribution” points (approximately 0.14% and 0.21% of all input grid points in the Ab21 and PDGF38 test sets, respectively) with attribution magnitude greater than 1σ (one standard deviation of the attribution point distribution in test set molecules, see Methods) from the zero-attribution baseline.

### Positive attribution patches cover large surface areas in very high viscosity antibodies

**Figure 3:**
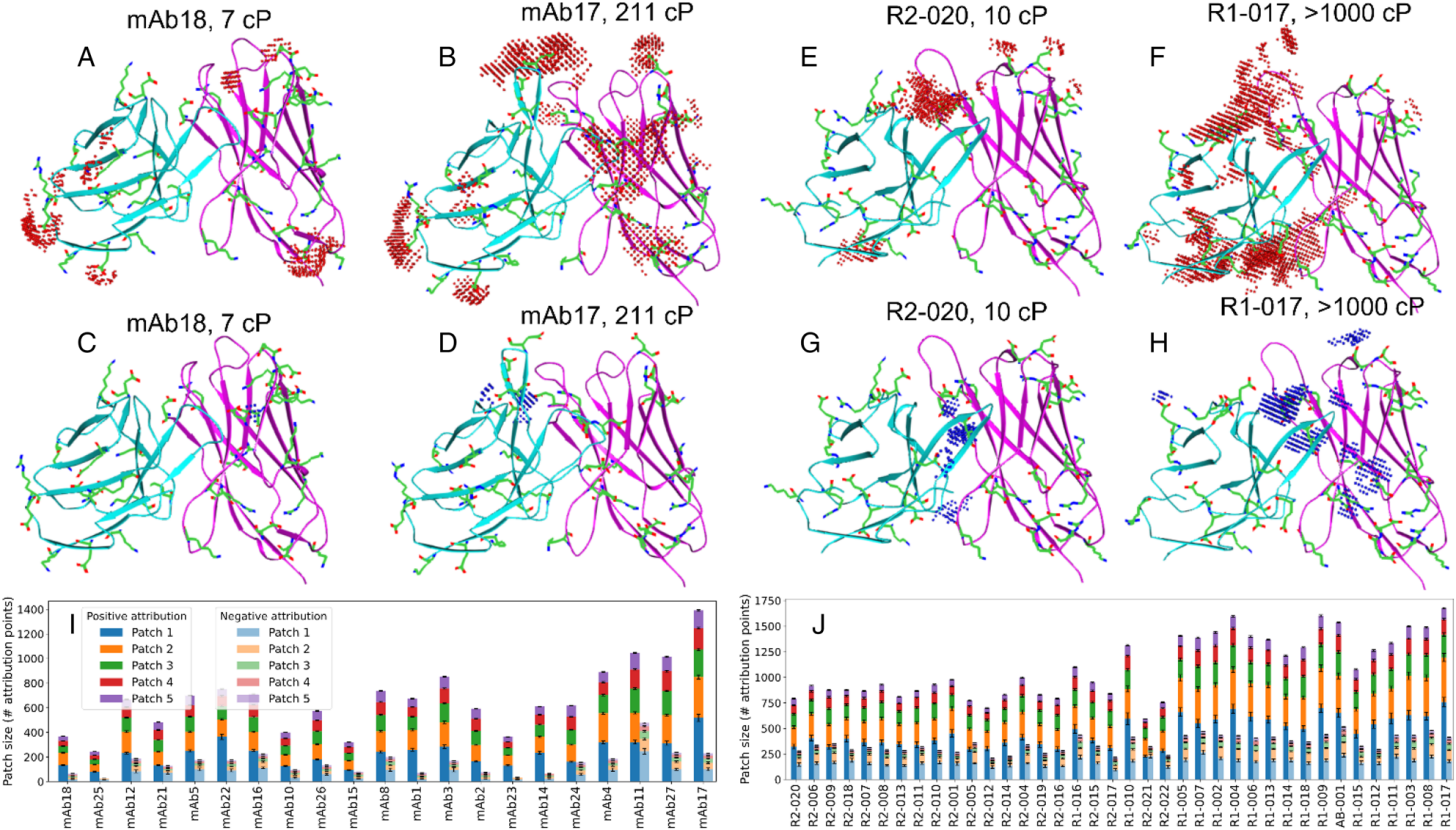
PfAbNet feature attribution maps and patch-size distributions in test set molecules. (**A**–**H**) Attribution maps and the variable region structure of four antibodies in the Ab21 (**A**–**D**) and PDGF38 (**E**–**H**) sets. The grid points with “significant attribution” (absolute attribution score greater than one standard deviation from the zero-attribution baseline) are shown. The light and heavy chain of each Fv structure are shown in cyan and magenta, respectively. Separate depictions of positive- (red dots, top row) and negative- (blue dots, bottom row) attribution maps highlight the greater size and density of the positive attribution map compared to the negative attribution map in each molecule. These examples were selected to illustrate the differences between the lowest- and highest-viscosity molecules in the Ab21 (**A** vs. **B**, **C** vs. **D**) and PDGF38 (**E** vs. **F**, **G** vs. **H**) sets. The contrast between the positive attribution maps of the highest- and lowest-viscosity (**B** vs. **A**, **F** vs. **E**) antibodies is particularly notable. (**I**,**J**) Patch-size distributions of up to five largest positive- and negative-attribution patches (contiguous segments of significant attribution grid points) in Ab21 (**I**) and PDGF38 (**J**). To highlight the dependence of patch size on measured viscosity, the antibodies in these panels are arranged based on their experimental viscosity, lowest to highest. The error bars represent the 95% confidence interval estimated using an ensemble of 100 predictions for each test set antibody (“Methods”).

Our analysis based on visual inspections of the attribution maps in the context of the corresponding Fv domain structures shows that (1) test set antibodies contain several spatially distinct surface patches, composed of attribution points with predominantly positive or negative scores, (2) positive-attribution patches in a molecule are more common and cover wider surface area compared to negative-attribution patches and (3) higher viscosity antibodies generally contain greater number of positive-attribution points that span over wider surface area compared to lower viscosity molecules. This is illustrated in Fig. 3 by attribution maps and underlying variable region structure of the lowest- and highest-viscosity antibodies in the Ab21 and PDGF38 sets.

Further quantitative analysis of attribution maps confirmed the trends we observed by visual inspection. The dependence of viscosity on positive-attribution patch areas, as shown in Fig. 3I,J, where bars are arranged according to the experimental viscosity, is particularly notable. This trend is more prominent in the Ab21 set, where the five highest-viscosity antibodies (η > 90 cP, five rightmost bars in Fig. 3I) have substantially larger attribution patch area compared to the other lower-viscosity (η < 25 cP) antibodies in this set (991 vs. 565 attribution points, p-value 7e−4). In the case of the PDGF38 set, however, since all, except 4 antibodies, exhibit high viscosity, this trend is not as definitive as in the Ab21 set. Nonetheless, the same subset of highest-viscosity PDGF38 antibodies (η > 90 cP, bars on the right half in Fig. 3J) also have substantially higher patch area relative to the other lower-viscosity antibodies (bars on the left half in Fig. 3J) in this set (1328 vs. 876 attribution points, p-value 5e−8). Thus, the patch-area analysis presented in Fig. 3I,J strongly suggests that specific structural and chemical motifs contribute to increasing viscosity and they are more likely to be present in high-viscosity antibodies.

### Proximal positive charges significantly reduce sidechain carboxyl attributions

**Figure 4:**
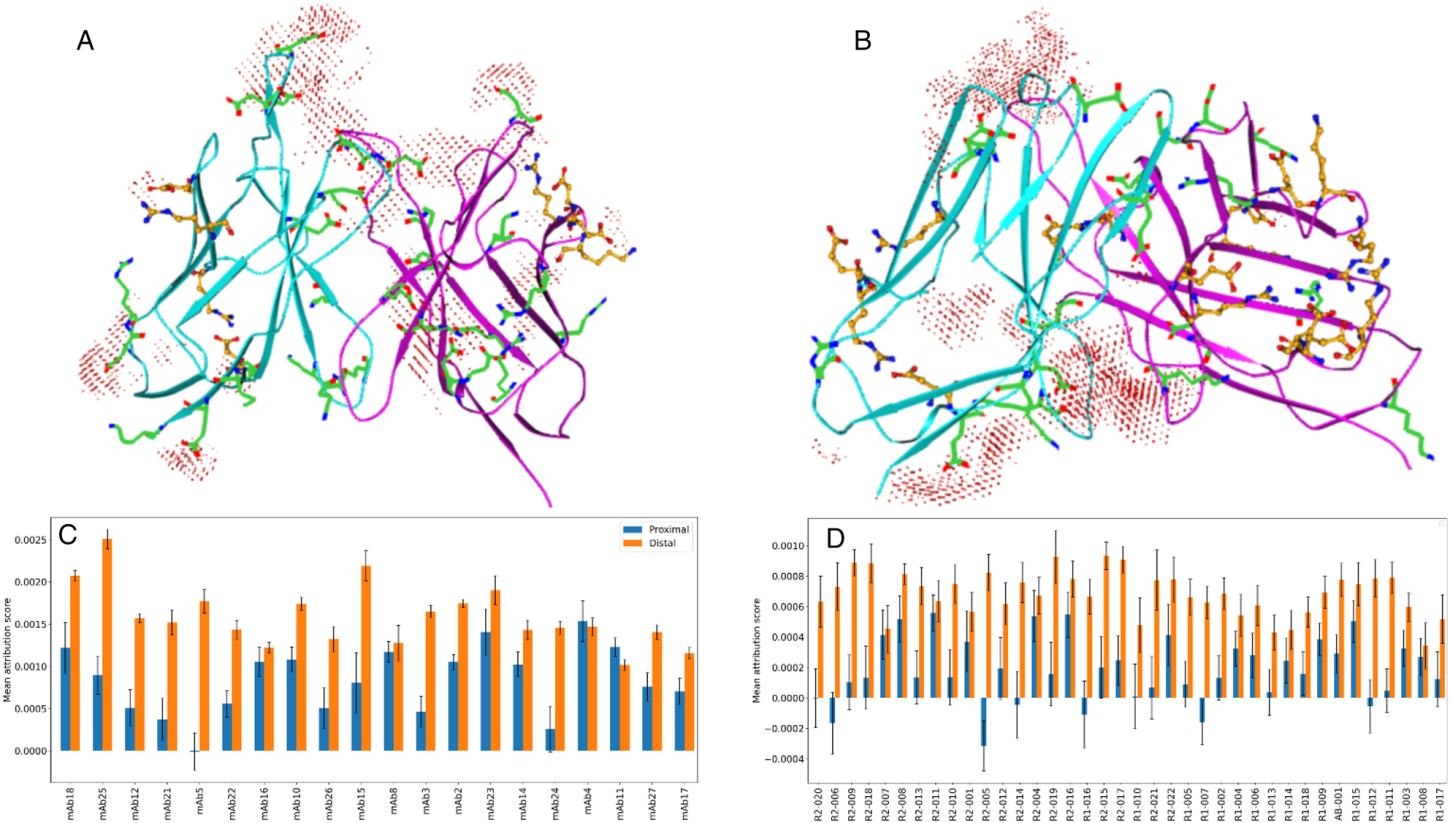
Influence of proximal positive charges on positive attributions around sidechain carboxyl groups. (**A**,**B**) Positive-attribution map and variable region structure of the highest-viscosity antibody in Ab21 (**A**) and PDGF38 (**B**) set. The light and heavy chain of each Fv structure are shown in cyan and magenta, respectively. The effect of proximal positive charges on the attribution maps is highlighted by orange, ball-and-stick depiction of relevant amino acids. (**C**,**D**) Average attribution score of Asp/Glu carboxylates in the proximity of (proximal, d ≤ 3.5 Å) or away from (distal, d ≥ 5 Å) a positive charge center (positively charged nitrogen in Lys or the Guanidine group in Arg) in Ab21 (**C**) and PDGF38 (**D**). The error bars represent the 95% confidence interval estimated using an ensemble of 100 predictions for each test set antibody.

Our qualitative analysis further showed that positive-attribution patches often overlap with sidechain carboxyls. However, the attribution around the sidechain carboxyls of some negatively charged residues is either negligible or is completely missing, as illustrated by the attribution maps of two highest-viscosity antibodies in Ab21 and PDGF38 sets (Fig. 4). In particular, we note that the carboxyl groups that are in the vicinity of a positive charge center generally receive little positive attribution.

We examined the generality of this observation by comparing the average attribution score of carboxyl grid points that are in the proximity of a positive charge (proximal set, d ≤ 3.5 Å) vs. those that are farther away (distal set, d ≥ 5 Å). For each test set molecule, we calculated the average attribution score of the proximal and distal set (see Methods). A striking contrast between the average attribution score of the two sets can be seen (Fig. 4C,D). The carboxyl groups in the distal set of nearly all test set antibodies have significantly greater average positive attribution compared to those in the proximal set, where the average attribution score is either significantly closer to zero or in many cases it is negative. This analysis clearly demonstrates that positive charges have a strong neutralizing effect that reduces positive attributions due to the nearby Asp/Glu carboxylates and that introduction of such positive charges can be an effective viscosity reduction strategy, as demonstrated recently^23^.

### Key structural and biophysical determinants of high viscosity

To determine the relative importance of different variable region segments, we calculated the contribution of the framework region and each CDR loop to the largest positive-attribution patches. Our analysis shows that positive-attribution patches are not localized to any particular Fv segment but are distributed throughout the framework and CDR loop regions (Fig. S4).

**Figure 5:**
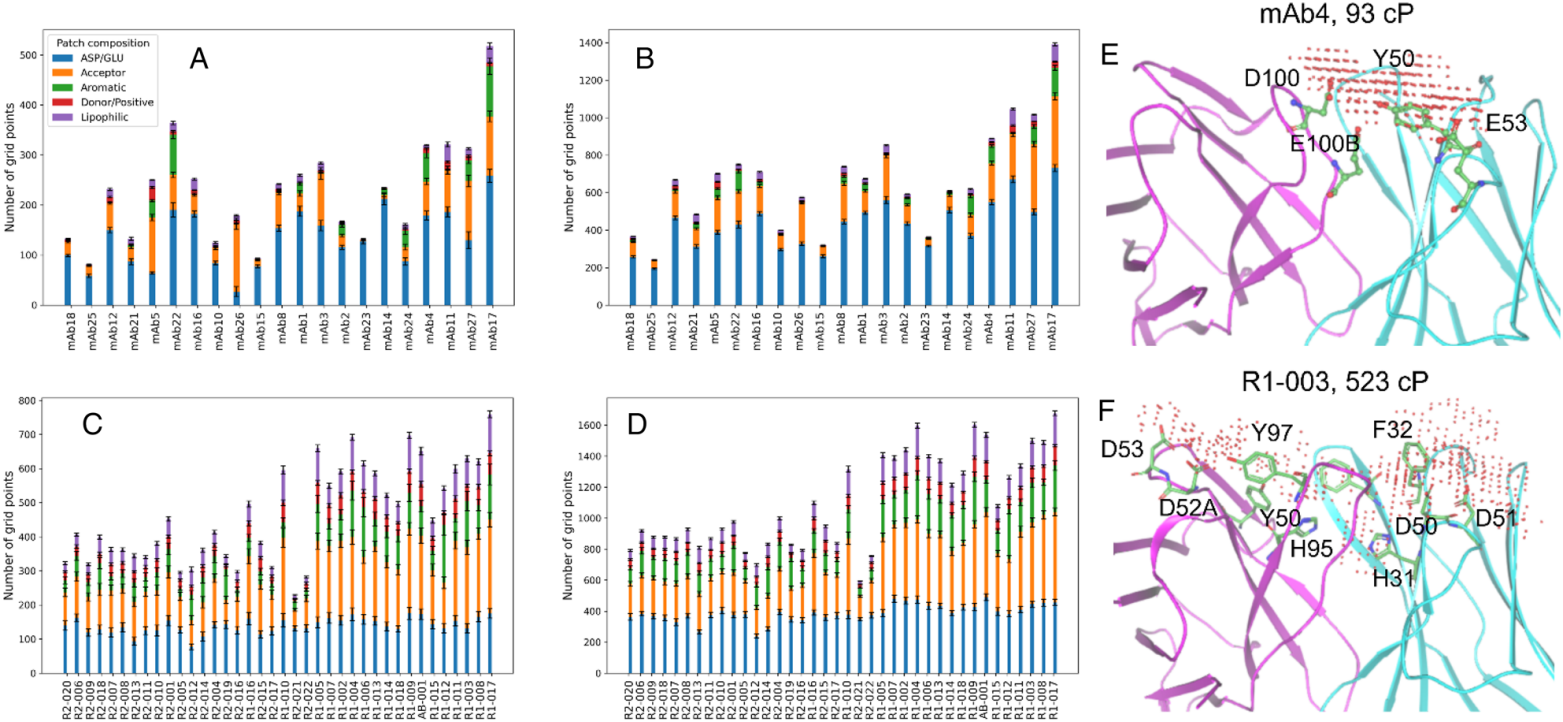
Key biophysical determinants of high viscosity. (**A**–**D**) The composition of the largest (**A**,**C**) and the five largest (**B**,**D**) positive-attribution patches in Ab21 (**A**,**B**) and PDGF38 (**C**,**D**). (**E**,**F**) The largest positive-attribution patch and the variable region structure of two high-viscosity antibodies from Ab21 (**E**, mAb4) and PDGF38 (**F**, R1-003). Negatively charged amino acids at either ends of each patch combine with the nearby surface aromatic residue(s) to form large contiguous attribution patches. The light and heavy chain of each Fv structure are shown in cyan and magenta, respectively. The error bars represent the 95% confidence interval estimated using an ensemble of 100 predictions for each test set antibody.

We performed similar analysis to determine the relative importance of the following biophysical features to the largest positive-attribution patches in the test set antibodies: (1) Asp/Glu sidechain, (2) hydrogen bond acceptor, (3) aromatic sidechain, (4) hydrogen bond donor and positive charge groups, and (5) lipophilic (see Methods). The contribution of each feature to the largest and the five largest positive-attribution patches in the Ab21 and PDGF38 antibodies are shown in Fig. 5.

We found that two features, Asp/Glu sidechain and hydrogen bond acceptor together make the majority of the contribution to the positive-attribution patch areas in the test set antibodies, with 89% of all positive attributions in the Ab21 and 68% in the PDGF38 set. Furthermore, the mainchain carbonyl oxygens contribute to nearly 73% of all attributions due to acceptors (Fig. S5). Since the negatively charged residues along with the acceptor atoms with negative partial charges largely define the negative electrostatic surface, these findings are consistent with the previous studies^9,14,15,23,24^ that have identified negative charge patches as the major determinant of antibody viscosity.

We also note significant aromatic content in the positive-attribution patches of high-viscosity antibodies. In the Ab21 set, the average aromatic content of the positive-attribution patches in the 5 highest-viscosity antibodies (η > 90 cP) is nearly 10% (last five bars on the x-axis of Fig. 5A,B). vs. 4% in the rest of the lower-viscosity antibodies (η < 25 cP). We also observe substantial aromatic content in the positive-attribution patches of nearly all PDGF38 antibodies (16% average aromatic content), most of which exhibit high viscosity (Fig. 5C,D). It is therefore reasonable to infer that the presence of aromatic residues would have a role in imparting high viscosity.

A potential molecular basis of how aromatic residues can exert such influence on viscosity can be understood by analyzing the positive-attribution patches in the context of the underlying Fv structure. Figure 5 shows two examples of high-viscosity antibodies [mAb4 (93 cP) and R1-003 (523 cP)], where aromatic residues make substantial (10% in mAb4 and 17% in R1-003) contribution to the largest positive-attribution patch. The attribution patch on both antibodies covers a wide area at the interface of the light and the heavy chain, with negatively charged amino acids at the opposite ends of both patches, which are separated by > 10 Å in mAb4 and > 20 Å in R1-003. Another common feature in these two examples is the presence of aromatic residues in the region between the flanking Asp/Glu residues. In the mAb4 example, a tyrosine residue (L-Y50) on the light chain separates the negatively charged residues on either end of the attribution patch (L-E53 from H-D100 and H-E100B). The attribution patch on R1-003, on the other hand, overlaps with a cluster of surface aromatic residues (H-Y97, H-Y50, H-H95, L-H31, L-F32) that separate two pairs of negatively charged residues at the opposite end of the attribution patch (L-D50 and L-D51; H-D52A and H-D53). Thus, the two examples presented here point to a common mechanism where surface aromatic sidechains enable the formation of a large contiguous negative patch by connecting the distant negative charge patches due to Asp or Glu and therefore might contribute to the observed high viscosity in these molecules.

### Models trained on surface hydrophobicity input are less generalizable

To assess the role of hydrophobicity, which has been described an important contributor to high-concentration viscosity in previous publications^13,15,29^, we trained additional models using a 3D grid representation of the Fv surface based on Eisenberg hydrophobicity scale^13,15,30^ of the underlying surface atoms (see “Methods”). Unlike the ESP grid, the Eisenberg grid comprised two channels, separately mapping the contributions from hydrophobic (positive Eisenberg scale) and hydrophilic (negative Eisenberg scale) surface atoms. As shown in Fig. S6, the generalization accuracy of the models trained with Eisenberg grid representation were worse than those trained using ESP input. The performance further degraded when the models were trained on a combined ESP-Eisenberg representation (cubic grid with 3 channels). While the performance gap between the ESP- and Eisenberg-based models can partly be attributed to how the PfAbNet architecture was originally developed and refined with ESP input, these results nevertheless demonstrate the importance of electrostatics over hydrophobicity-based features in describing the high concentration viscosity behavior of mAbs in our datasets.

### Sensitivity to ESP representation settings, Fv conformational variability, and data augmentation

We tested the performance of our models by exploring different settings for the ESP grid resolution and the surface shell thickness. As expected, model showed better performance when the network was trained with higher resolution ESP grid (Fig. S7). However, the model performance is less sensitive to the surface shell thickness (Fig. S8).

Since high-throughput screening of early-stage mAbs, even before any materials are available, is an important potential application of our method, we tested the impact of conformational variability in the Fv domain homology models on PfAbNet predictions. As shown in Fig. S9 and further discussed in Supplementary Note 2, the relative prediction variability due to alternate Fv input conformations is generally small, which gives us the confidence that structural variations in different homology models will likely have only limited impact on any prioritization and selection of early-stage antibodies based on the PfAbNet models.

We also assessed the impact of data augmentation on model performance by generating up to 10 samples by randomly rotating each dataset antibody at training and inference. As shown in Fig. S10, we obtained better or comparable performance when models were trained with 10 × augmentation and predictions were generated using an ensemble of 10 randomly rotated structure for each antibody compared to the other lower values for augmentation and ensemble sizes that we explored.

## Discussion

Recognizing the importance of electrostatics as a key driver of antibody viscosity from previous studies, we chose the surface ESP map as the only input to our neural network, PfAbNet. This input representation restricts the network to focus only on the most important surface characteristics of the antibody variable region, masking less relevant structural details that could lead to overfitting. Moreover, as demonstrated by our sensitivity analysis, surface representations are generally less sensitive to conformational variability in homology models, which further helps improve generalization accuracy and practical utility of our approach.

The PfAbNet generalization performance demonstrated here is particularly notable because these models were trained on only a few dozen training examples, whereas the number of network parameters exceeded the training set by over 4–5 orders of magnitude. Furthermore, the network was trained only with the given ESP surface representation of the Fv region, without incorporating any prior knowledge about any sequence- or structure-based features that are known to be relevant for viscosity^14,15^. Nonetheless, the network was able to learn how features such as negative electrostatics surface patches as well as positive charge centers around those patches influence the high concentration viscosity behavior of antibodies.

Although the role of negative charges on antibody viscosity has been recognized and mitigation strategies based on the removal of such charge patches have been successfully applied^24^, mutation of Asp and Glu can also disrupt important electrostatic interactions with the antigen and can result in significant loss of binding affinity. Based on the importance of surface aromatic sidechains that can enlarge negative electrostatic patch, as identified by our attribution analysis, we believe that removing bridging aromatic residues, in some cases, can be an alternate strategy for reducing negative electrostatic patch area, and therefore, viscosity.

While this study confirms the importance of electrostatics in determining antibody viscosity behavior, the absence of similar association with hydrophobicity could either be dataset dependent or a more nuanced analysis may be needed to understand their role on viscosity. Protein–protein interaction (PPI) and reversible self-association, which has been shown to correlate with viscosity^25,29^, is known to be driven by a complex interplay of electrostatics and hydrophobic interactions, requiring specific spatial organization of charge centers and hydrophobic surface patches at the interface^31,32^. Attribution analysis presented in this work could be further extended to investigate whether similar arrangements of surface ESP patches and intensities, representing distribution of charges and hydrophobicity, might also be a driver of high-concentration antibody viscosity.

Since access to large datasets is limited for many biological problems, a major challenge in applying machine learning in this field has been to develop generalizable models that can be trained under a low-N setting. Recent publications^33,34^ utilizing embeddings generated from protein language models^34–36^, trained on large corpus of protein sequence data, have shown that a high-level of generalization can be achieved in a limited training data regime. However, the work presented here demonstrates for the first time that a deep learning model, with p > > n can be trained from scratch to produce high-level of generalization.

Geometric deep learning^37,38^, an emerging technique that has recently been utilized to learn protein surface representations^39,40^, could be used as an alternative to the end-to-end 3D-CNN architecture presented here. However, unlike 3D-CNN, considerable time and effort is required to identify a relevant set of input features for training geometric deep learning models. Nonetheless, this method could be applied to the current problem to assess its generalization performance in a low-data regime.

The surface ESP representation, a key feature of PfAbNet, is not specific to antibodies and can be applied to other tasks where surface properties play important role, e.g., prediction of DNA-binding sites on DNA-binding proteins. We believe that our method can provide a more accurate prediction for this and similar tasks by capturing the ESP surface features in much greater detail compared to the existing methods that utilize pre-computed structure-based^41^ or sequence-based^42,43^ descriptors.

## Methods

### Dataset

The heavy and light chain sequences of Ab21 antibodies and the corresponding experimental viscosity at 150 mg/mL concentration were obtained from the previous study by Lai^12^. The PDGF38 sequences and the corresponding measured viscosity at the same 150 mg/mL concentration were obtained from an earlier publication by Apgar^23^. Although the viscosity profiles of the antibodies in these two sets were measured at slightly different pH (6.0 for Ab21 vs. 5.8 for PDGF38), our results demonstrates that models trained on Ab21 can be meaningfully evaluated on the PDGF38 set, and vice versa.

### Structure modeling and ESP representation

The Fv domain models were generated using Bioluminate software package (2021-2 release, Schrodinger LLC, New York). A single homology model for each sequence in our data set was generated using the default Bioluminate settings. By default, the models were generated with the Chothia numbering scheme. The sensitivity analysis of PfAbNet to input Fv conformations was based on 10 homology models that were generated using the “-nmodel 10” option in Bioluminate.

For each homology model representing an Fv region, a 3D grid representing the electrostatic potential surface was generated using the following procedure: (1) The coordinates of the protein were moved so that its center of mass was located at the cartesian origin, (2) the Connolly^44^ molecular surface was generated using OpenEye Spicoli toolkit (release 1.5.2.1, OpenEye Scientific Software, Santa Fe, NM) using 0.75 Å grid resolution and 1.4 Å probe radius, (3) the ESP was calculated using the Poisson-Boltzmann^45^ method from OEZap toolkit (release 2.4.1.1, Openeye Scientific Software, NM), using OPLS_2005^46^ charges with inner and outer dielectric constants of 1 and 80, respectively, (4) the ESP around the molecular surface was mapped onto a cubic grid ranging from − 36 Å to + 36 Å along each Cartesian axis with a uniform spacing of 0.75 Å between grid points. Grid points located inside the molecular surface, where ESP is ill-defined, were masked. Additionally, grid points located outside, but > 2 Å distance from the molecular surface were also masked. This resulted in a surface shell of approximately 2 Å thickness with non-zero ESP at grid points located within this shell; all other masked grid points were assigned a numerical value of zero.

We adopted commonly used techniques (e. g. Poisson-Boltzmann) and parameter settings (e. g. probe sphere, dielectric constants, and charges) to generate the input ESP grids. However, the grid spacing and the surface shell thickness were determined by independently exploring various reasonable values of these parameters. The parameter settings that produced the most performant models (Figs. S7 and S8) were selected.

### 3D convolutional neural network

3D-CNN models were defined and trained using the PyTorch^47^ (version 1.10.0) deep learning framework. The network comprised 6 convolutional layers, each with 3 × 3 × 3 kernel, followed by a rectified linear activation unit (ReLU) and a max pooling layer. The first convolution layer comprised 4 filters. The number of filters in each successive layer was doubled and the max pooling operation reduced the spatial dimensionality by half. The output of the final convolution layer is flattened to produce a 1024-dimensional feature vector, which is then passed through a drop out layer with a dropout^48^ rate of 0.05, and finally to the output layer comprising a single node. The network weights were initialized using the Glorot^49^ scheme. The network was trained by minimizing Huber loss using ADAM^50^ optimizer with default parameters. The models were trained with a batch size 1 and a fixed learning rate of 10^–5^.

### Hyperparameter tuning

As noted earlier, the PfAbNet architecture was developed using only our internal data. Hyperparameter tuning was performed to train the most accurate model, as measured by the Spearman rank-order correlation and ROC-AUC, across the leave-group-out test sets, where training/test split was done based on the therapeutic program. Individual parameters, including learning rate, batch size, model depth, number of convolutional filters, kernel size, pooling type, and weight decay, were adjusted iteratively by sampling commonly accepted range of values for these parameters. Learning rate was sampled between 0.01 and 10^–6^ at equally separated points that differed by an order of magnitude. Batch size values of 1, 2, 4, 8, 16, and 32 were explored. Model architectures that increased the feature maps by a factor of 2 after each successive layer were explored, sampling the following feature map sizes for the first layer: 2, 4, 8, 16, and 32. The depth of the model was varied between 2 and 8 convolution blocks. Alternate architectures where the feature map size was initially increased for the first few layers than gradually decreased over the next successive layers were also explored. Models with the following kernel size for the convolutional layers were trained: 3, 5, 7, 9, and 11. Networks containing L2 regularization of convolutional layers with weight decay values of 10^–2^, 10^–3^, and 10^–4^ were trained. Max and average pooling layers with kernel size of 2 and 4 were used as part of the hyperparameter tuning exercise. Model architectures comprising fully connected layers of varying width with number of nodes between 10 and 500 were explored.

### Data augmentation

We used data augmentation to overcome the challenges posed by insufficient training data. Each starting Fv homology model was randomly rotated, generating 10 samples for each training data point. The coordinates of each resulting structure were then used to generate the ESP grid. This data augmentation approach provides an additional benefit for the current task as it can potentially mitigate the well-known issue of instability in ESP calculations due to their sensitivity to the separation between charge centers and grid points. The same data augmentation procedure was also applied at inference and predictions from each rotated structures were averaged to produce viscosity of the test molecule.

### PfAbNet training and inference

The network was trained from scratch, generating two separate PfAbNet models that we refer to as: (1) PfAbNet-PDGF38 (model trained on the PDGF38 set) and (2) PfAbNet-Ab21 (model trained on the Ab21 set). We trained additional models, referred to as PfAbNet-LOOCV, to test leave-one-out cross-validation performance, where each Ab21 antibody is left-out once as the test set while the model is trained on the remaining 58 antibodies (38 from the PDGF38 and 20 from the Ab21 set). The PDGF38 antibodies were excluded from this LOOCV test because high sequence similarity within this set will result in artificially high leave-one-out prediction accuracy for these antibodies. Whereas the Ab21 antibodies are significantly diverse (Fig. 1L) and, therefore, a LOOCV performance on this set is expected to be a good indicator of the generalization performance of our network.

Each of the three PfAbNet models (PfAbNet-Ab21, PfAbNet-PDGF, and PfAbNet-LOOCV) refer to an ensemble of 10 models, each trained using tenfold cross-validation split of the data into training and validation sets. In each case, the network was trained for 2000 epochs and the model with best validation loss in the last 50 epochs of the training was saved for evaluation.

The inference for each test set antibody was obtained using an ensemble of 10 structures that were generated through random rotation of the starting Fv structure, as described above. Each structure in the ensemble was then used as input to each of the 10 cross-validation PfAbNet models, thus generating a total of 100 predictions for each test set molecule. The final prediction was taken as the average of these 100 predictions.

### PfAbNet inference time

Given a 3D structure or a model of an antibody variable region, the PfAbNet end-to-end pipeline takes approximately 10 min using an Intel Xeon CPU core and an Nvidia Tesla V100 GPU. Since molecular surface and ESP calculations take nearly all the compute time, a 10 × higher throughput can be achieved simply by running these calculations on each rotation-augmented structure in parallel on a multi-core workstation that are commonly used in research settings today.

### Model interpretation by integrated gradients attribution

The Integrated Gradients implementation of PyTorch Captum library was used to compute attribution of predicted viscosity with respect to each point in the input ESP grid. We used the PfAbNet-PDGF and PfAbNet-Ab21 models to calculate attribution grid for each antibody in the corresponding left-out test sets, Ab21 and PDGF38, respectively. To gain meaningful insights, we focus our analysis on a subset of highest magnitude attribution points, calculating separate “significant attribution” thresholds for PfAbNet-PDGF and PfAbNet-Ab21 models. To determine significant attribution threshold for a given model, we first combined the predicted non-zero attribution scores from each antibody in the corresponding test set. The standard deviation of the resulting distribution was taken as the significant threshold for that model. Using this approach, we obtained the significant threshold of the PfAbNet-PDGF and PfAbNet-Ab21 models as 4.1e−4 and 3.7e−4, respectively. Figure S11 shows the distribution of attribution scores of Ab21 and PDGF38 antibodies.

Each antibody contains several spatially distinct patches of attribution points. A patch was defined as a contiguous segment of significant attribution points such that each constituent point was within 1.5 Å (twice the grid resolution) from at least one another point in that patch.

The biophysical feature composition of each patch was determined by assigning each constituent attribution points to one of the following category, based on the type of the nearest protein atom: (1) Asp/Glu (any sidechain atom from these negatively charge amino acids), (2) hydrogen bond acceptor (any Oxygen, except the Asp/Glu carboxylate), (3) aromatic (any sidechain atom from His, Phe, Tyr, or Trp residue), (4) hydrogen bond donor (any Nitrogen, except those that are part of any previous category) and Lys/Arg (any atom from the Lys amino or the Arg Guanidine group), or (5) lipophilic (any Carbon atom, except those included in any other category).

The same procedure was applied to assign each grid point to a Fv segment: the framework region or a CDR loop. Subsequently, the composition of the largest and the five largest attribution patches on each test set antibody to different Fv segments were calculated.

The impact of proximal positive charges on sidechain carboxyl attributions was calculated using the following procedure. For each test set antibody, we first created a proximal and a distal set, each comprising non-overlapping subset of Asp and Glu residues in the molecule. These two sets were constructed based on the distance between the carboxylate and the nearest positive charge center (positively charged nitrogen atom in Lys or the Guanidine group in Arg): proximal (${d}_{min,caboxylate-cation}\le 3.5$ Å) and distal (${d}_{min,caboxylate-cation}\ge 5$ Å). Next, for each set (proximal and distal), we calculated an average attribution score from the attribution score of grid points associated with the sidechain carboxyl atoms in each group. The association between a grid point and the corresponding carboxylate was made using the following two criteria: (1) the closest protein heavy atom to that grid point was part of the carboxyl motif and (2) the minimum distance between the grid point and at least one of the three carboxyl atom was less than 4.0 Å.

### Representation of Eisenberg hydrophobicity and hydrophilicity

An Eisenberg representation was described by two separate cubic grids, hydrophobic and hydrophilic, each with the same dimension and grid spacing as used for ESP grid. Each grid was constructed by mapping either the hydrophobic or the hydrophilic atomic densities onto the grid points. The sign of Eisenberg hydrophobicity scale^30^ was used to classify an amino acid as hydrophobic or hydrophilic. Starting from a given Fv structure, both types of grid were generated using a similar procedure, as described here for the hydrophobic grid: (1) hydrophilic amino acids (residues with negative Eisenberg scale) were removed, (2) van der Waals radius of each remaining atom was set to 3 × the Eisenberg scale (absolute value) of the parent amino acid, (3) density at each grid point was calculated as a linear sum of the contribution from each atom-centered Gaussian in the molecule, where each Gaussian was described by the same height, but the width was determined by the atomic radius (Eisenberg scale) set in step 2, and (4) similar to the ESP representation, grid points that were located either inside the molecular surface or those that were located > 2 Å distance outside of the surface were masked, setting the value of those grid points to zero.

## Supplementary Information

Supplementary Information.

## Data Availability

The datasets generated and/or analyzed during the current study are either available in the PfAbNet-viscosity github repository or can be reproduced using the code and Jupyter Notebooks available in this repository. pfizer-opensource/pfabnet-viscosity (github.com).

## Code availability

Code for PfAbNet model training and inference as well to reproduce all analysis and figures presented in this work is available at pfizer-opensource/pfabnet-viscosity (github.com).
