# Supplementary material for: Low-data interpretable deep learning prediction of antibody viscosity using a biophysically meaningful representation
Source: Sci Rep. 2023 Feb 20;13:2917. doi: 10.1038/s41598-023-28841-4 (PMC9941094; doi:10.1038/s41598-023-28841-4)
Supplement: Supplementary file 1 — Supplementary Information. [file 41598_2023_28841_MOESM1_ESM.docx]

Low-Data Interpretable Deep Learning Prediction of Antibody Viscosity using a Biophysically Meaningful Representation

Brajesh K Rai^1*^, James Apgar^2^, and Eric M. Bennett^2^

^1^Machine Learning and Computational Sciences, ^2^Biomedicine Design, Pfizer Worldwide Research Development and Medical, 610 Main Street, Cambridge, Massachusetts 02139, United States

KEYWORDS: antibody, viscosity, deep learning, convolutional neural network, ESP

ABSTRACT: Deep learning, aided by the availability of big data sets, has led to substantial advances across many disciplines. However, many scientific problems of practical interest lack sufficiently large datasets amenable to deep learning. Prediction of antibody viscosity is one such problem where deep learning methods have not yet been explored due to the relative scarcity of relevant training data. In this work, we overcome this limitation using a biophysically meaningful representation that enables us to develop generalizable models even under limited training data. We present, PfAbNet-viscosity, a 3D convolutional neural network architecture, to predict high-concentration viscosity of therapeutic antibodies. We show that with the electrostatic potential surface of the antibody variable region as the only input to the network, the models trained on as few as couple dozen datapoints can generalize with high accuracy. Our feature attribution analysis shows that PfAbNet-viscosity has learned key biophysical drivers of viscosity. The applicability of our approach to other biological systems is discussed.

**Supplementary Note 1:** **Classification performance of PfAbNet-Ab21 vs null model on test sets with more balanced composition of low- and high-viscosity antibodies**

Classification performance of PfAbNet-Ab21 relative to null model was further evaluated on test sets that were created by down-sampling different number of high-viscosity antibodies from the combined PDGF38 and Ab8 set. Specifically, we randomly selected high-viscosity antibodies and combined those with the low-viscosity data points in PDGF38 and Ab8 to generate the following two groups: 1) 50 test sets comprising high- and low-viscosity antibodies in 2:1 ratio and 2) 50 test sets with equal number of high- and low-viscosity antibodies. The bar plots D and E in Fig. S3 show the average classification accuracy of PfAbNet-Ab21 and the null model from the 50 test sets in each group. Note that the null model used in this evaluation assigns all test set antibodies to the high-viscosity class.

**Supplementary Note 2:** **Sensitivity to the conformational variability of the input homology models**

For each test set antibody, we generated 10 homology models and the corresponding PfAbNet predictions. As shown in Fig. S9, the prediction variability is generally small, with a low median 𝜎/𝜇 (standard deviation over prediction mean) ratio: 0.34 for the PDGF38 set and 0.37 for the Ab21 set. While a subset of high-viscosity PDGF antibodies have high 𝜎, the 95% confidence interval for 80% predictions remain in the same viscosity class as the prediction mean, which suggests that the choice of a low-energy conformation from a homology model ensemble as input to PfAbNet may not be critical to its prediction performance. The relatively low 𝜎/𝜇 ratio for most test set antibodies also gives us the confidence that structural variations in different homology models will likely have only limited impact on any prioritization and selection of early-stage antibodies based on the PfAbNet models.

Table S1. Experimental viscosity (at 150 mg/mL) and PfAbNet-Ab21 prediction of PDGF38 antibodies.

| **Entity** | **Expt. viscosity**  **(150 mg/mL)** | **Prediction** |
| --- | --- | --- |
| AB-001 | 440 | 240.3 |
| R1-002 | 288 | 146.4 |
| R1-003 | 523 | 165.6 |
| R1-004 | 310 | 174.2 |
| R1-005 | 190 | 147.0 |
| R1-006 | 314 | 133.9 |
| R1-007 | 233 | 115.0 |
| R1-008 | 567 | 112.5 |
| R1-009 | 430 | 202.9 |
| R1-010 | 99 | 92.8 |
| R1-011 | 519 | 206.6 |
| R1-012 | 471 | 84.3 |
| R1-013 | 414 | 101.4 |
| R1-014 | 415 | 92.1 |
| R1-015 | 452 | 97.7 |
| R1-016 | 73 | 60.6 |
| R1-017 | 1000 | 178.1 |
| R1-018 | 416 | 97.7 |
| R2-001 | 37 | 29.1 |
| R2-004 | 54 | 30.2 |
| R2-005 | 37 | 15.8 |
| R2-006 | 13 | 21.4 |
| R2-007 | 21 | 22.0 |
| R2-008 | 23 | 27.0 |
| R2-009 | 19 | 19.3 |
| R2-010 | 35 | 30.6 |
| R2-011 | 26 | 20.4 |
| R2-012 | 39 | 13.0 |
| R2-013 | 26 | 19.0 |
| R2-014 | 51 | 21.6 |
| R2-015 | 83 | 25.3 |
| R2-016 | 67 | 21.9 |
| R2-017 | 84 | 29.7 |
| R2-018 | 20 | 22.3 |
| R2-019 | 60 | 24.8 |
| R2-020 | 10 | 21.4 |
| R2-021 | 119 | 10.8 |
| R2-022 | 135 | 27.8 |

Table S2. Experimental viscosity (at 150 mg/mL) and PfAbNet-PDGF38 and PfAbNet-LOOCV predictions of Ab21 antibodies.

| **Entity** | **Expt. viscosity**  **(150 mg/mL)** | **PfAbNet-Ab21**  **prediction** | **PfAbNet-LOOCV**  **prediction** |
| --- | --- | --- | --- |
| mAb1 | 14.4 | 73.6 | 34.2 |
| mAb2 | 20.9 | 67.2 | 23.6 |
| mAb3 | 14.9 | 120.2 | 34.6 |
| mAb4 | 93.4 | 115.7 | 42.9 |
| mAb5 | 8.6 | 71.8 | 39.6 |
| mAb8 | 12.9 | 124.7 | 45.0 |
| mAb10 | 10.2 | 15.2 | 8.0 |
| mAb11 | 100.3 | 351.8 | 92.9 |
| mAb12 | 7.5 | 31.0 | 17.7 |
| mAb14 | 23.4 | 106.0 | 58.9 |
| mAb15 | 12.9 | 11.4 | 6.2 |
| mAb16 | 10.0 | 65.7 | 26.3 |
| mAb17 | 210.9 | 1564.5 | 269.5 |
| mAb18 | 7.1 | 12.4 | 7.2 |
| mAb21 | 8.6 | 29.5 | 11.3 |
| mAb22 | 9.0 | 28.7 | 20.5 |
| mAb23 | 22.9 | 16.5 | 8.8 |
| mAb24 | 90.5 | 89.3 | 32.7 |
| mAb25 | 7.4 | 7.6 | 4.8 |
| mAb26 | 10.3 | 47.4 | 17.8 |
| mAb27 | 103.8 | 307.7 | 90.1 |

Table S3. Experimental viscosity (at 150 mg/mL) and PfAbNet-Ab21 predictions of Ab8 antibodies.

| **Entity** | **Expt. viscosity**  **(150 mg/mL)** | **PfAbNet-Ab21**  **prediction** |
| --- | --- | --- |
| TGN1412 | 16.42 | 13.7 |
| Basiliximab | 25.05 | 14.2 |
| Natalizumab | 13.67 | 20.4 |
| Tremelimumab | 8.80 | 4.1 |
| Ipilimumab | 8.60 | 8.0 |
| Atezolizumab | 11.56 | 19.5 |
| Ganitumab | 10.10 | 18.4 |
| Vesencumab | 23.57 | 5.8 |

Table S4: Performance of PfAbNet and the previous models, measured using coefficient of determination (R^2^).

|  |  | Coefficient of determination (R^2^)^^^^ | | | | |
| --- | --- | --- | --- | --- | --- | --- |
| Training set (N) | Test set (N) | Sharma^^^ | SCM | PfAbNet | p-value | |
|  |  |  |  |  | PfAbNet vs. Sharma | PfAbNet vs. SCM |
| Ab21 (21) | PDGF38 (38) | 0.49 (0.03) | 0.60 (0.03) | 0.62 (0.02) | 2e-83 | 5e-2 |
| PDGF38 (38) | Ab21 (21) | 0.64 (0.1) | 0.40 (0.07) | 0.75 (0.08) | 2e-25 | 6e-141 |
| PDGF38 + Ab21 (LOOCV) (58) | Ab21 (21) | 0.48 (0.08) | 0.40 (0.08) | 0.79 (0.08) | 7e-78 | 1e-107 |

^^^Performance on each test set was evaluated using parameters that were derived by fitting to the corresponding training set data; ^^^^values in parentheses represent the 95% confidence interval based on bootstrap standard error


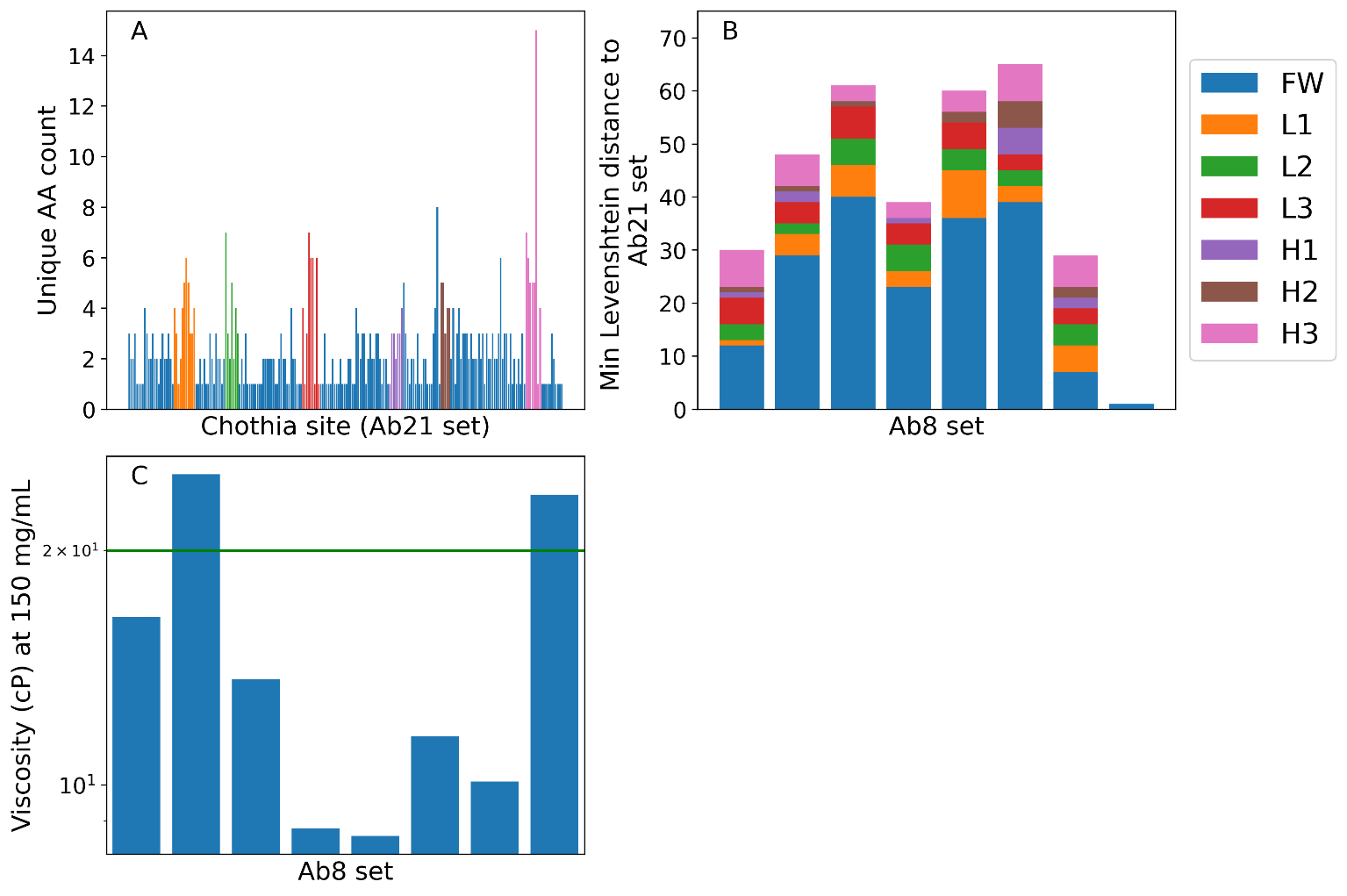


**Fig. S1. Ab8 dataset.** **A** Amino acid variability in the Ab8 dataset at different Chothia positions across the variable region sequence. **B** Minimum Levenshtein distance between the variable region sequence of the Ab8 antibodies with respect to the Ab21 set. **C** Experimental viscosity of the Ab8 antibodies at 150 mg/mL concentration.


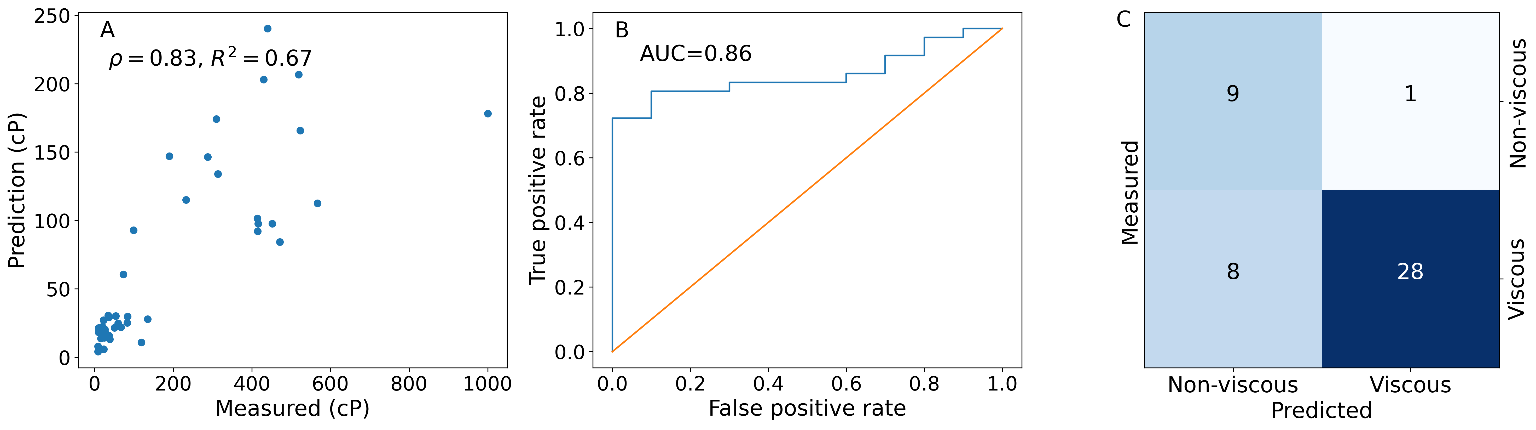


**Fig. S2. Performance of PfAbNet-Ab21 on the test set comprising PDGF38 and Ab8 antibodies.** All predictions and experimental values correspond to viscosity at 150 mg/mL concentration. **A** PfAbNet-Ab21 predictions vs. experimental values for the PDGF38 and Ab8 antibodies. **B**, **C** Classification performance: ROC curve (**B**) and confusion matrix (**C**). The confusion matrix was calculated using the optimal operating point, derived from the ROC curve in **B**, as the cutoff for viscous vs. non-viscous class.


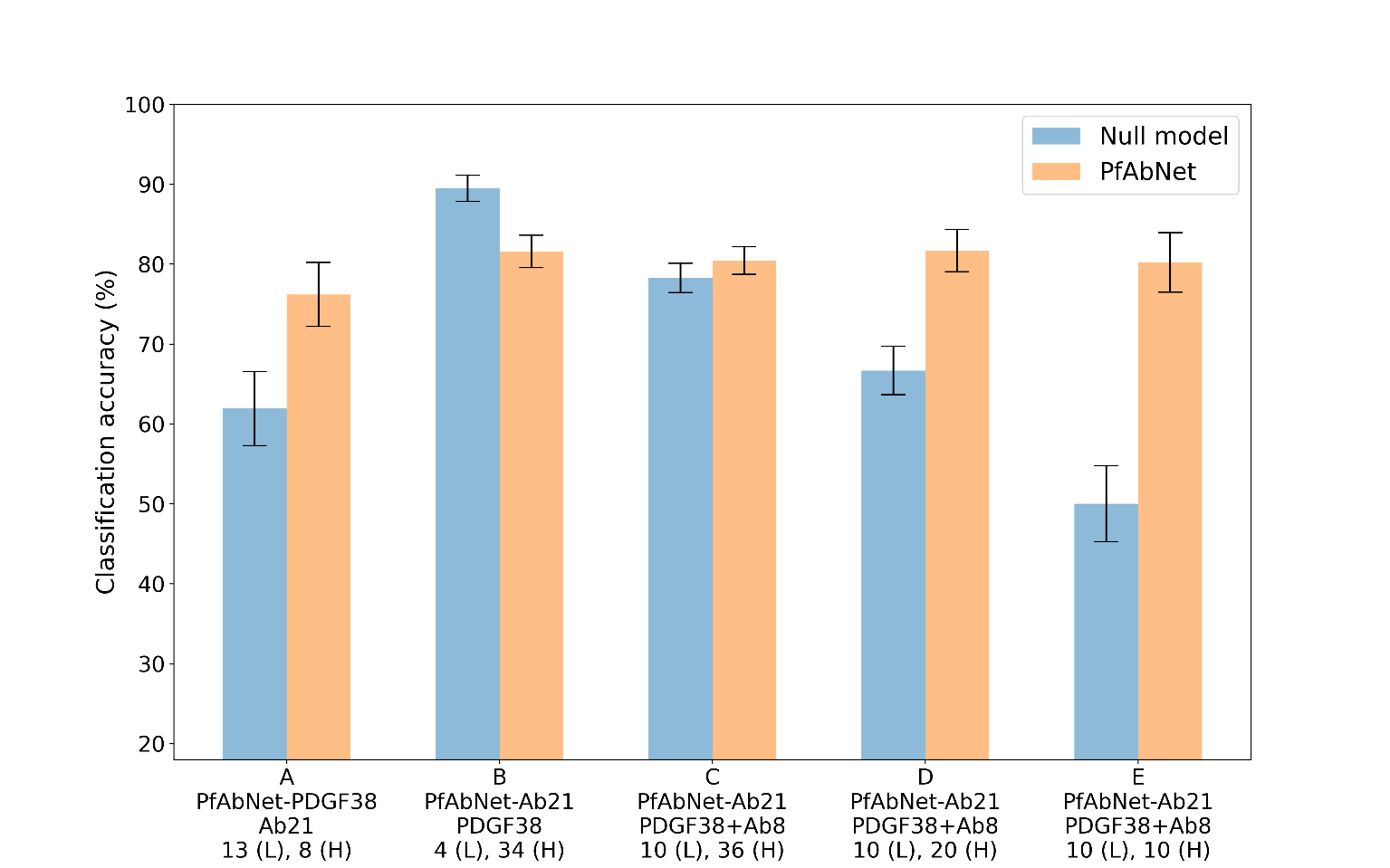


**Fig. S3. Classification accuracy of PfAbNet relative to Null models.** **A** Classification accuracy on the Ab21 test set was evaluated using PfAbNet-PDGF38 and a null model that assigned all test set antibodies to the low-viscosity class. **B-E** PfAbNet-Ab21 and a null model that assigned all test set antibodies to the high-viscosity class were used to assess classification accuracy on the PDGF38 (**B**), test set comprising PDGF38 and Ab8 antibodies (**C**), and test sets comprising subsets of PDGF38 and Ab8 that were obtained by down-sampling the high-viscosity antibodies in this set, as described in Supplementary Note 1 (**D**, **E**). The last row under each bar shows the number of low- (L) and high-viscosity (H) antibodies in the corresponding test set. Classification accuracy of the PfAbNet models were calculated using optimal operating point as the cutoff to separate the low- from the high-viscosity class.


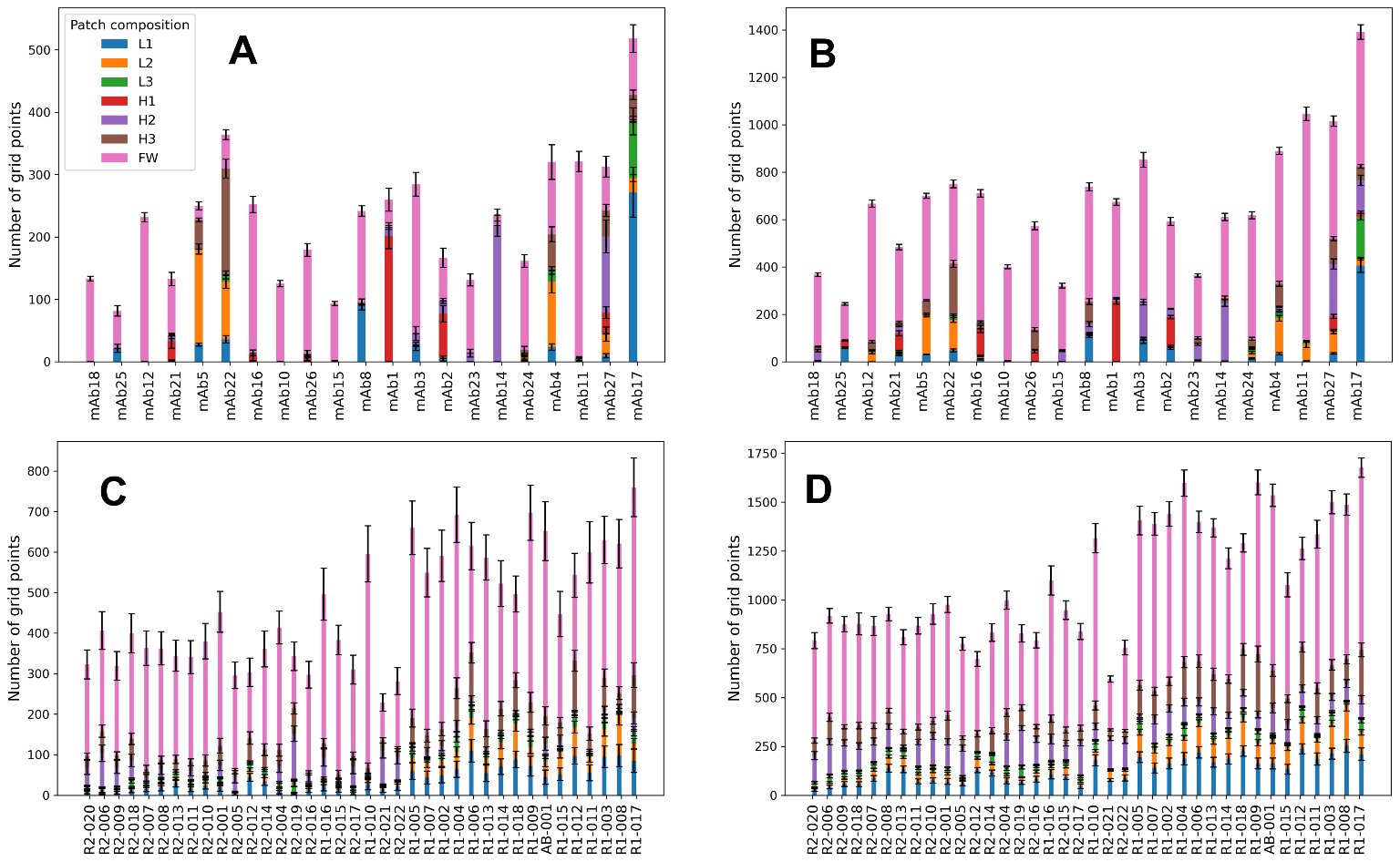


**Fig. S4. Distribution of attribution patches by structural segments.** Contribution of the framework and CDR regions to the largest (**A** and **C**) and the five largest (**B** and **D**) attribution patches in the Ab21 (**A** and **B**) and PDGF38 (**C** and **D**) antibodies. The error bars represent the 95% confidence interval based on an ensemble of 100 predictions for each test set antibody.


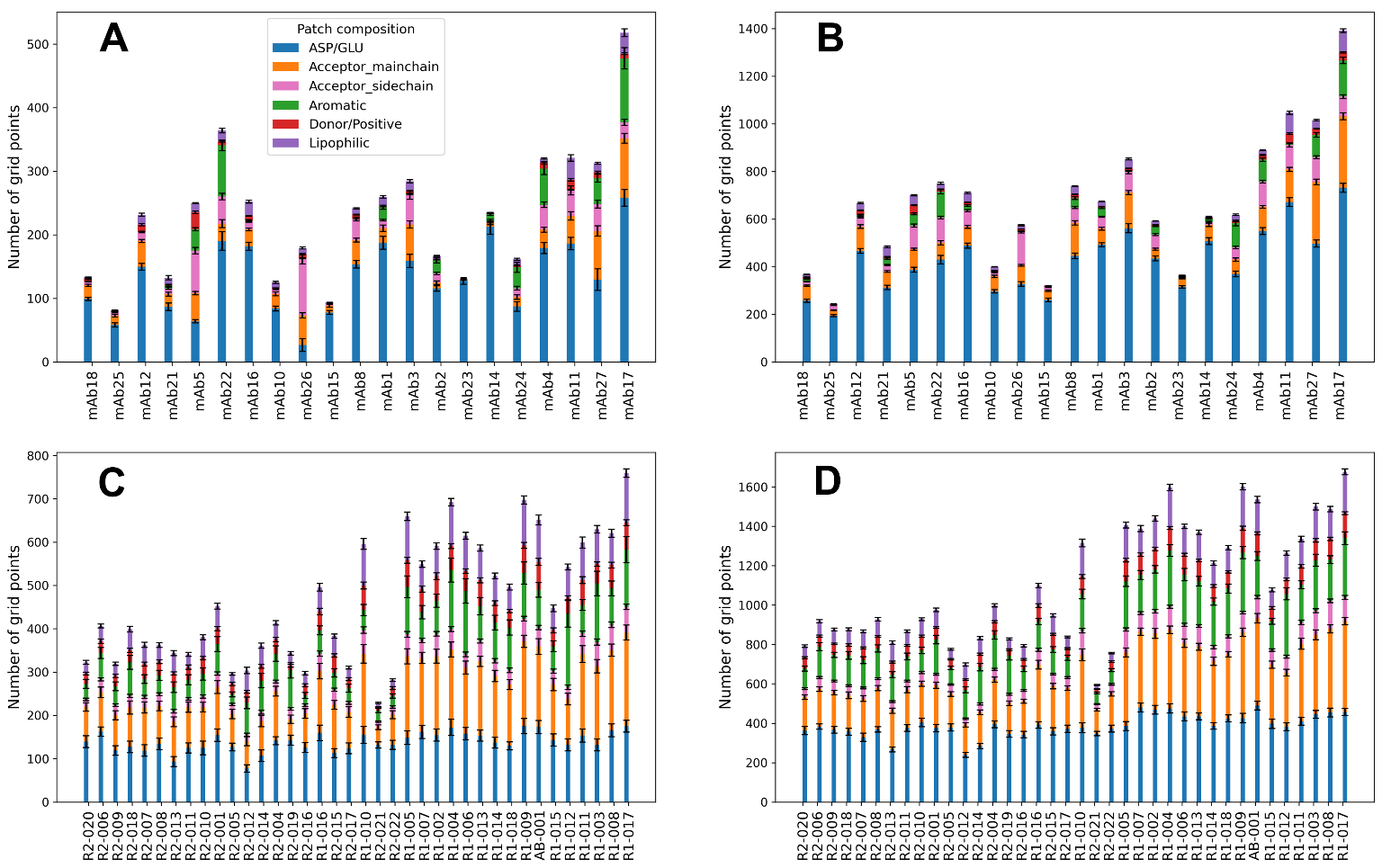


**Fig. S5. Chemical composition of attribution patches.** The chemical composition of the largest and the five largest attribution patches in Ab21 (**A** and **B**) and PDGF38 (**C** and **D**). The distributions displayed in this figure is similar to that shown in Fig. 5 (main text), except that the contribution from acceptors were further split into two components by separately calculating contributions from: 1) the mainchain carbonyls and 2) the sidechain oxygens.


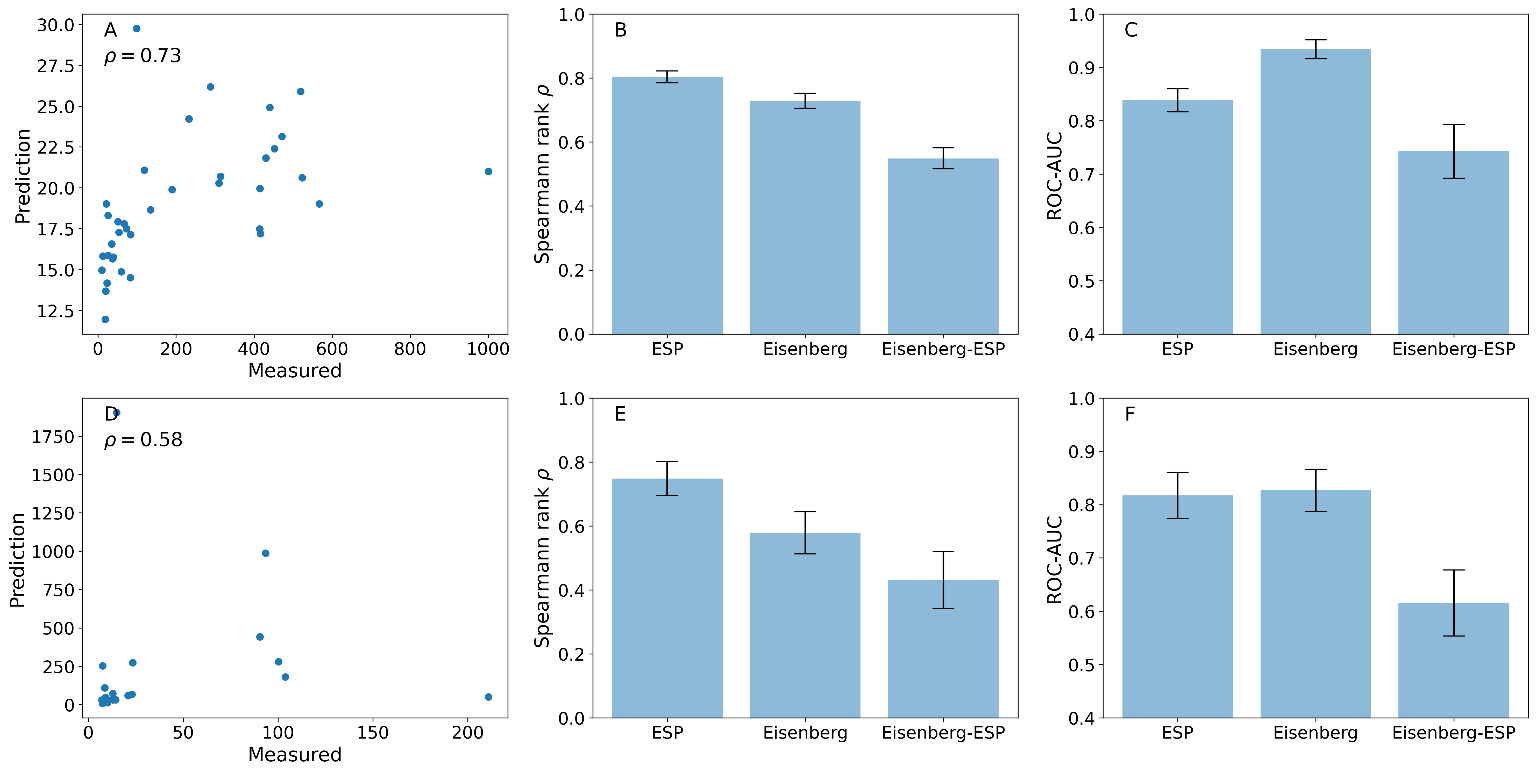


**Fig. S6. Sensitivity to input representation.** Performance of PfAbNet models trained with different input grid representations: ESP, Eisenberg (2 channels, representing hydrophobic and hydrophilic surface shell), and Eisenberg-ESP (3 channels, combining ESP and Eisenberg grids). The 3D-CNN models using Eisenberg or Eisenberg-ESP representation were trained from scratch using the same dataset and the training procedures as used to train ESP-based models. **A** PfAbNet-Ab21 (Eisenberg) predictions on the PDGF38 test set. **B**, **C** Regression (**B**) and classification (**C**) performance of PfAbNet-Ab21 on the PDGF38 test set using different input grid representation. **D** PfAbNet-PDGF (Eisenberg) predictions on the Ab21 test set. **E**, **F** Regression (**E**) and classification (**F**) performance of PfAbNet-PDGF on the Ab21 test set using different input grid representation. The error bars represent the 95% confidence interval based on bootstrap standard error.


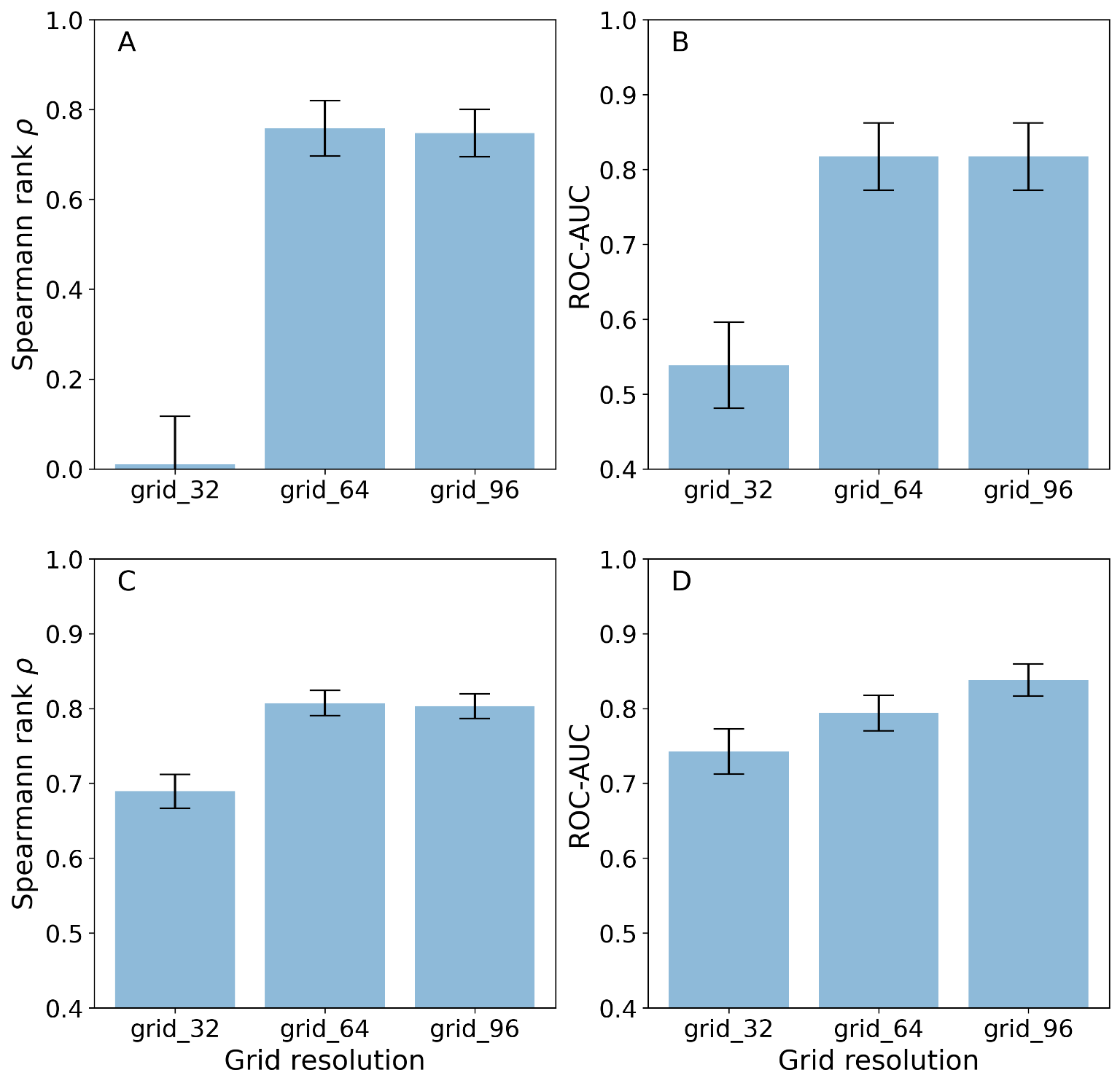


**Fig. S7. Sensitivity to grid resolution.** PfAbNet performance using different grid resolution settings for the input ESP representation. The input ESP grids were generated using three different settings, grid_N, where N=32, 64, 96 represents the number of grid points along each dimension of the cubic grid with the corresponding resolution of 2.5 Ǻ, 1.25 Ǻ, and 0.75 Ǻ, respectively. **A**, **B** Regression (**A**) and classification (**B**) performance of PfAbNet-Ab21 model on the PDGF40 test set. **C**, **D** Regression (**C**) and classification (**D**) performance of PfAbNet-PDGF model on the Ab21 test set. The error bars represent the 95% confidence interval based on bootstrap standard error.


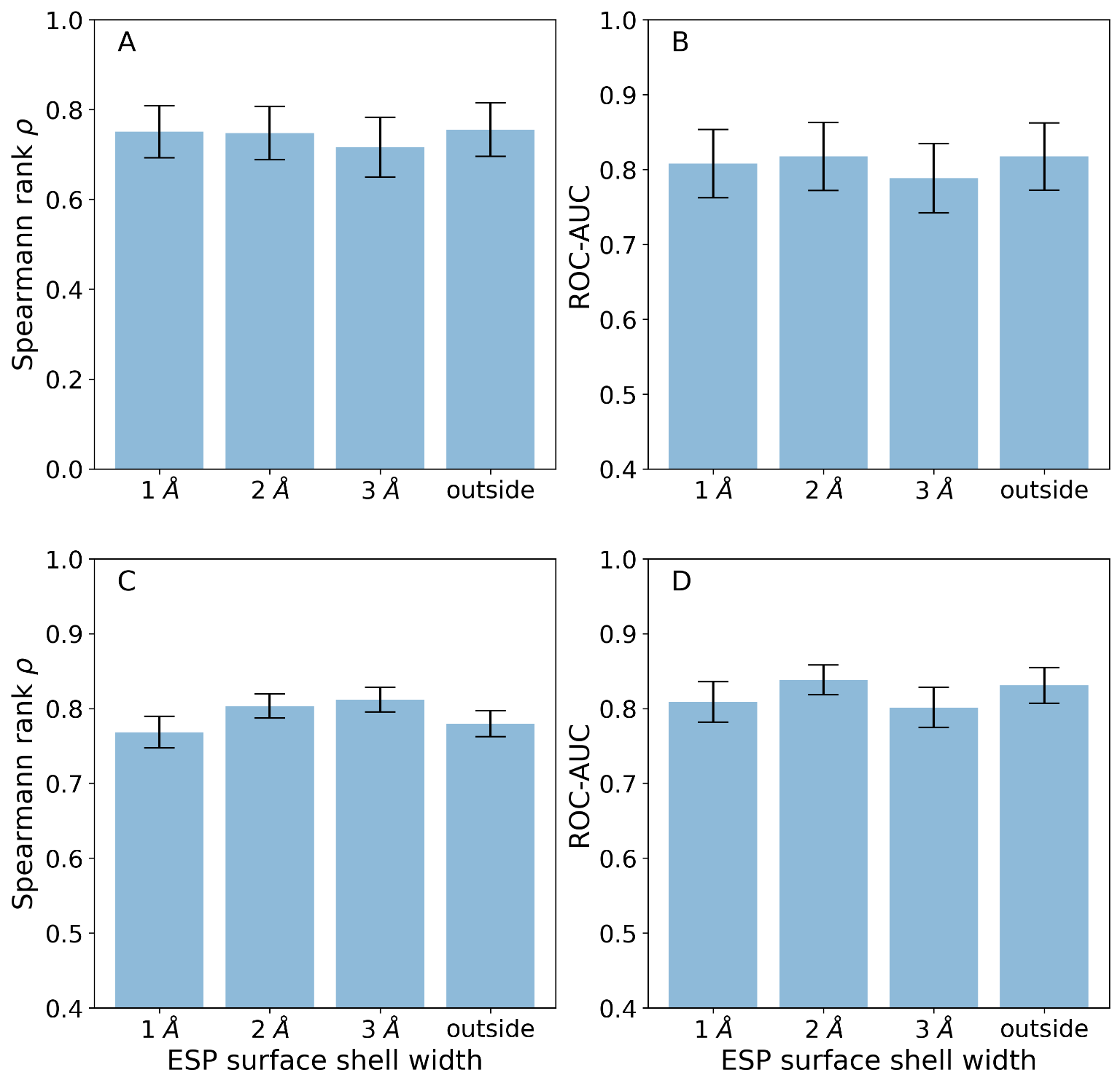


**Fig. S8. Sensitivity to ESP surface shell width.** PfAbNet performance using different masking distance threshold to represent the input ESP grid: 1 Ǻ, 2 Ǻ, 3 Ǻ. All grid points beyond the specified distance were masked. No masking was applied in the case of the “outside” category. **A**, **B** Regression (**A**) and classification (**B**) performance of PfAbNet-Ab21 model on the PDGF38 test set. **C**, **D** Regression (**C**) and classification (**D**) performance of PfAbNet-PDGF model on the Ab21 test set. The error bars represent the 95% confidence interval based on bootstrap standard error.


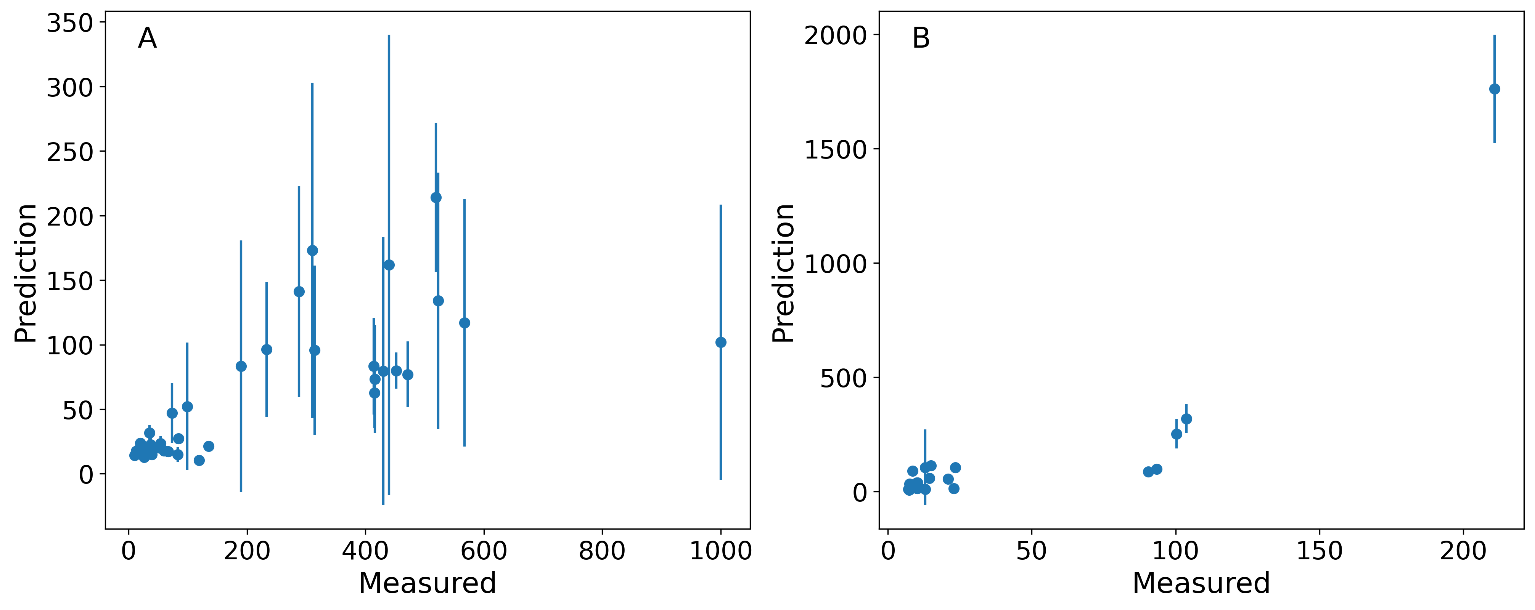


**Fig. S9. Sensitivity to input Fv conformation variability.** **A** PfAbNet-Ab21 predictions on the PDGF38 test set. **B** PfAbNet-PDGF predictions on the Ab21 test set. The error bars represent the 95% confidence interval based on the predictions from 10 different input homology models.


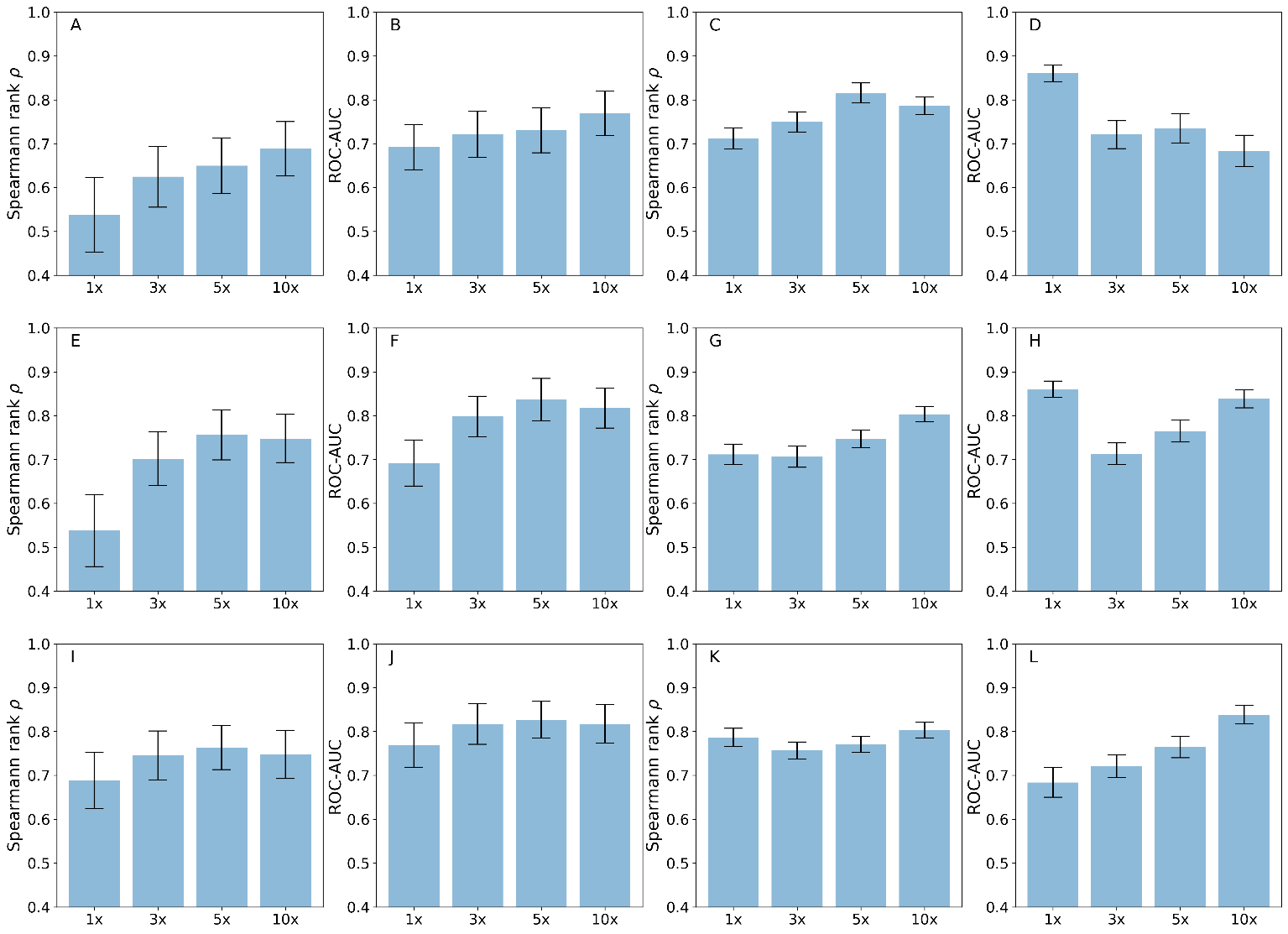


**Fig. S10. Sensitivity to data augmentation.** PfAbNet performance with different levels of training data augmentation and inference ensemble size. The training and test set structures were generated through random rotation of each Fv structure N times (N = 1, 3, 5, or 10). (Top row) **A**-**D** Effect of training data augmentation on the regression and classification performance of PfAbNet-Ab21 on PDGF38 (**A** and **B**) and PfAbNet-PDGF on Ab21 (**C** and **D**). (Middle row) **E**-**H** Effect of varying inference ensemble size on the regression and classification performance of PfAbNet-Ab21 on PDGF38 (**E** and **F**) and PfAbNet-PDGF on Ab21 (**G** and **H**). (Bottom row) **I**-**L** Effect of training data augmentation and inference ensemble size on the regression and classification performance of PfAbNet-Ab21 on PDGF38 (**I** and **J**) and PfAbNet-PDGF on Ab21 (**K** and **L**). The error bars represent the 95% confidence interval based on bootstrap standard error.


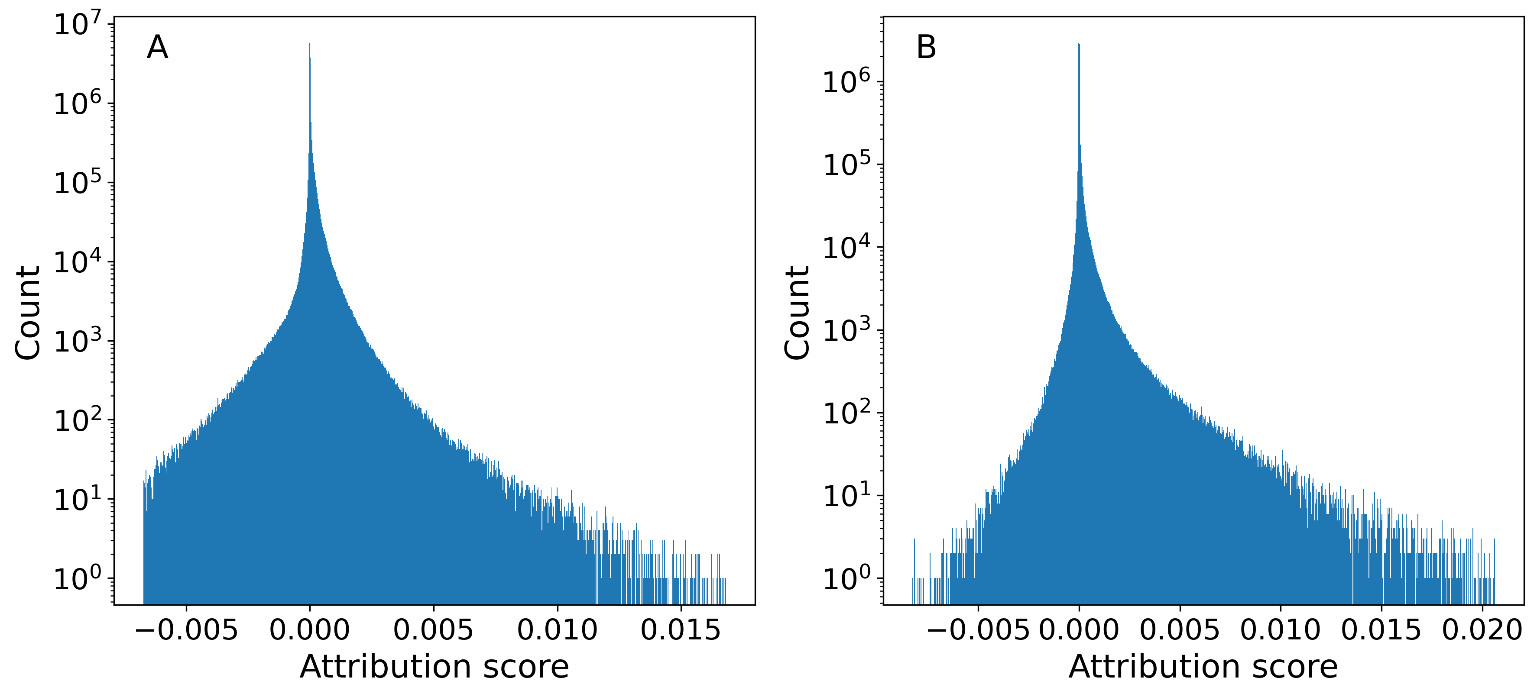


**Fig. S11. Distribution of attribution scores.** The attribution scores were generated using **A** PfAbNet-Ab21 model on the PDGF38 test set and **B** PfAbNet-PDGF model on the Ab21 test set.
